# Emerging therapeutic options for follicular-derived thyroid cancer in the era of immunotherapy

**DOI:** 10.3389/fimmu.2024.1369780

**Published:** 2024-05-29

**Authors:** Naimah Turner, Sarah Hamidi, Rim Ouni, Rene Rico, Ying C. Henderson, Maria Puche, Sayan Alekseev, Jocelynn G. Colunga-Minutti, Mark E. Zafereo, Stephen Y. Lai, Sang T. Kim, Maria E. Cabanillas, Roza Nurieva

**Affiliations:** ^1^ Department of Immunology, The University of Texas MD Anderson Cancer Center, Houston, TX, United States; ^2^ Department of Endocrine Neoplasia and Hormonal Disorders, The University of Texas MD Anderson Cancer Center, Houston, TX, United States; ^3^ Department of Head and Neck Surgery, The University of Texas MD Anderson Cancer Center, Houston, TX, United States; ^4^ Department of Biology, College of Science and Engineering, Houston Christian University, Houston, TX, United States; ^5^ Program of Biology, College of Sciences, The University of Texas at San Antonio, San Antonio, TX, United States; ^6^ Program of Immunology, The University of Texas MD Anderson Cancer Center UTHealth Graduate School of Biomedical Sciences (GSBS), Houston, TX, United States; ^7^ Department of Rheumatology, Allergy and Immunology, Yale University, New Haven, CT, United States

**Keywords:** thyroid cancer, tumor immune microenvironment, targeted therapy, immunotherapy, mutational landscape

## Abstract

Although most follicular-derived thyroid cancers are well differentiated and have an overall excellent prognosis following treatment with surgery and radioiodine, management of advanced thyroid cancers, including iodine refractory disease and poorly differentiated/undifferentiated subtypes, is more challenging. Over the past decade, better understanding of the genetic drivers and immune milieu of advanced thyroid cancers has led to significant progress in the management of these patients. Numerous targeted kinase inhibitors are now approved by the U.S Food and Drug administration (FDA) for the treatment of advanced, radioiodine refractory differentiated thyroid cancers (DTC) as well as anaplastic thyroid cancer (ATC). Immunotherapy has also been thoroughly studied and has shown promise in selected cases. In this review, we summarize the progress in the understanding of the genetic landscape and the cellular and molecular basis of radioiodine refractory-DTC and ATC, as well as discuss the current treatment options and future therapeutic avenues.

## Introduction

**Figure 1:**
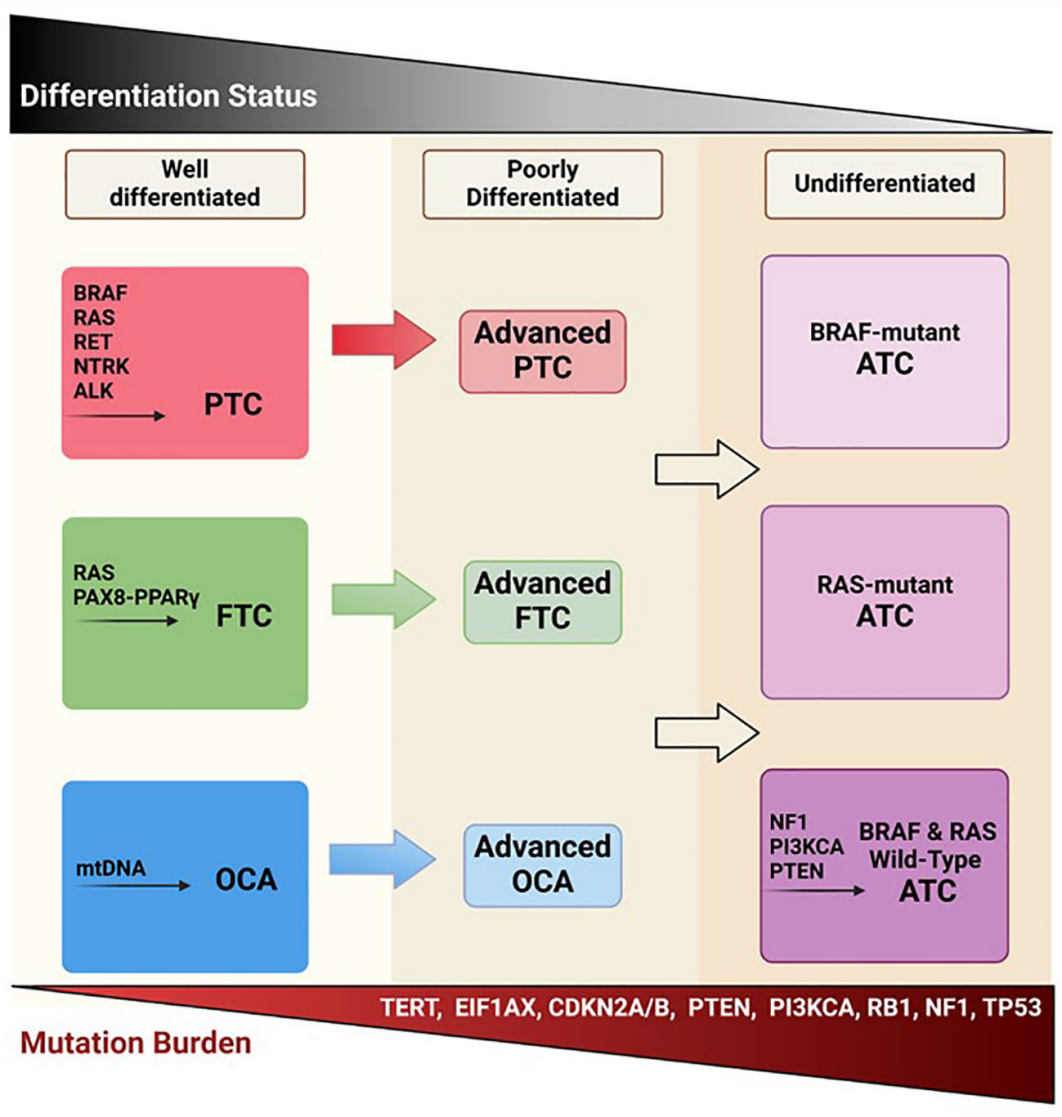
Follicular-derived thyroid cancer evolution. Well-differentiated TC histotypes (PTC, FTC, and OCA) are characterized by driver mutations in BRAF, RAS, RET, and mitochondrial DNA. It is thought that these DTCs, through the accumulation of various mutations, become less differentiated and more aggressive, potentially leading to ATC. Created with BioRender.com.

Thyroid cancer (TC) subtypes vary according to originating cell, histopathology, and clinical course. TCs arising from the thyroglobulin-producing follicular cells of the thyroid gland are classified based on their differentiation status, spanning from well-differentiated TCs (DTCs) to poorly differentiated (PDTC) (5% of TCs) or anaplastic (undifferentiated) (ATC) (1-2% of TCs) subtypes (1, 2). DTCs include papillary (PTC), follicular (FTC), and oncocytic carcinomas of the thyroid (OCA; formerly known as Hürthle cell TC), which account for about 90%, 4%, and 2% of all TCs respectively (3) (**Figure 1**). Aside from carcinomas originating from the follicular cells of the thyroid, medullary TC (MTC), derived from parafollicular cells (C-cells), accounts for 1-2% of TCs (3).

In the United States, TC is the 12^th^ most common cancer diagnosis overall and the 7^th^ most common for women, accounting for an estimated 44,000 new cases in 2022 (4, 5). TC incidence is nearly three times greater in women than men (4). In addition, the incidence rate increases with age, with an average age at diagnosis of 52 for women and 69 for men (4). Although incidence of this cancer has sharply increased over the last 3 decades, mortality rates have remained relatively low and stable at around 0.5 per 100,000 cases (4). While DTCs have a promising prognosis with a 98.4% 5-year relative survival, ATCs are more aggressive, with historical overall survival of only four to six months, accounting for 40-50% of all TC-related deaths in the United States (5, 6).

Although most DTCs have good overall outcomes and respond well to conventional treatment strategies such as surgery and radioactive iodine (RAI), 5-10% of patients will develop distant metastatic disease, which is often refractory to RAI (7). Additionally, ATC presents a unique clinical challenge to diagnose and treat effectively due to its rapid growth, highly metastatic nature, and relatively high mutational burden (6). Recent advances in molecular biology techniques have enabled deeper understanding of the genomic, cellular, and immunologic characteristics of advanced DTC and ATC, leading to FDA approval of several targeted therapies including sorafenib (DTC), lenvatinib (DTC), cabozantinib (second-line DTC), selpercatinib and pralsetinib (RET-alteredaltered TCs), as well as the combination of dabrafenib/trametinib (BRAFV600E mutated TCs) (8–13). Due to high expression of programmed death-1 (PD-1) and its ligand PD-L1 in more aggressive thyroid cancers, these carcinomas may benefit from therapies with immune checkpoint inhibitors (ICIs), especially ATC (14–16). Multiple studies have shown immunotherapy to be a promising option for patients with ATC (17–19). While much progress has been made, further study is needed to elucidate the mechanisms underlying ATC development in order to delineate novel predictive biomarkers and to improve treatment and survival outcomes for this fatal disease. In this review, we will focus on follicular-derived thyroid cancers, summarizing current understandings of their pathogenesis and the role of the immune system, contemporary treatment strategies, as well as future therapeutic perspectives.

## Clinical characteristics of TC

TC types vary by severity and are classified based on the TNM (Tumor, Node, Metastasis) staging system, which considers tumor size, lymph node status, and metastatic stage (31). Each subtype presents with unique clinical and cytomorphological features and can be differentially diagnosed via histopathological examination (32).

Papillary Thyroid Cancer (PTC) is a slow growing malignancy with the highest incidence of all TC subtypes, as it affects approximately 90% of patients with TC. Despite relatively high incidence, PTC generally has a better prognosis than any other subtype with a 5-year relative survival of 99% (4). PTC cells display larger, elongated nuclei with a clear appearance compared to the normally round follicular cell. Histological subtypes include follicular, tall cell, columnar, diffuse sclerosing and hobnail variants (33). Patients with PTC may present with a slow growing thyroid nodule and/or palpable cervical lymph nodes. In most cases, patients are asymptomatic. PTC has been found to metastasize to the lymph nodes primarily in the neck, as well as to the lungs (34, 35).

Follicular Thyroid Cancer (FTC) has a lower incidence than PTC at 4-5% of all TC cases and a slightly lower 5-year survival rate of 91-97.9% (3). FTC can be classified into minimally invasive, encapsulated angioinvasive, and widely invasive subtypes (36). Histological characteristics of FTC consist of enlarged and elongated nuclei, fibrotic scarring of the tumoral tissue, and an abundance of eosinophils in the lumen of the follicle (37, 38). Patients with FTC have higher rates of distant metastases (e.g. lung, bone, liver) than PTC and rarely metastasizes to the lymph nodes (39).

Oncocytic carcinoma of thyroid (OCA), previously known as “Hürthle cell” carcinomas, make up 1-2% of all TCs and have an average 5-year survival of 91% (40). OCAs usually present as an encapsulated tumor and are subclassified by the degree of capsular and/or vascular invasion into minimally invasive, encapsulated angioinvasive, and widely invasive subtypes (40). OCA is distinguished from other TC histotypes by an extensive presence of oncocytic cells (>75%) with eosinophilic cytoplasm caused by an abundance of dysfunctional mitochondria, a lack of nuclear features indicative of PTC, and high-grade features such as high mitotic activity and tumor necrosis (32, 41). OCA has been reported to be more prone to recurrence and metastasis than the non-oncocytic TCs (42). In fact, metastatic state has been reported to be an independent prognostic factor in OCA with distant metastatic disease significantly decreasing 5-year survival rates to 46% from 98.6% and 97.6% in local or regional disease, respectively (43).

### Poorly differentiated thyroid carcinoma and differentiated high-grade thyroid cancer

There has been much debate on how to classify carcinomas of the thyroid with intermediate prognoses and histological features falling between the classically differentiated TCs and the undifferentiated ATC. However, in the most recent fifth edition of the WHO classification, PDTC and DHGTC were recognized as two distinct types of high-grade non-anaplastic follicular cell-derived carcinomas. Tumors in both of these classes are known to often be large and highly invasive (32). DHGTC is a new intermediate entity where tumors retain the architectural and cytologic properties of the well-differentiated TCs but have a high mitotic rate and/or tumor necrosis is present. PDTCs are further on the dedifferentiation spectrum, characterized by solid, trabecular or insular growth in addition to tumor necrosis and/or high mitotic activity (32). PDTC and DHGTC are relatively rare subtypes of thyroid cancer, comprising about 1 to 6.7% of TCs and have a much poorer prognosis than well-differentiated TCs (32). DHGTC and PDTC have been reported to have similar 5-year disease specific survival rates of 68% and 70%, respectively (54).

Anaplastic Thyroid Carcinoma (ATC) is known for being the most aggressive form of TC with historically low survival and cure rates (6). It affects 1-2% of all TC patients in the United States. ATCs arise from previously well differentiated TC which acquire additional mutations, ultimately leading to anaplastic transformation (3, 55). Though ATCs are automatically classified as stage IV regardless of tumor burden or metastatic state, they are further subclassified according to locoregional and distant spread. Tumors confined to the thyroid gland are stage IVA, tumors with extrathyroidal extension and/or spread to regional lymph nodes are stage IVB, and tumors that have spread to distant sites outside the neck are stage IVC (56). Further highlighting ATCs’ aggressive nature, distant metastatic disease is seen in about 50% of patients at diagnosis (57). Common presenting symptoms of ATC include dysphonia, dysphagia, neck or ear pain, dyspnea, and weight loss (57). Morphological features of ATC include tissue invasion, high mitotic activity and necrosis (58).

## Mutational landscape of TC

**Figure 2:**
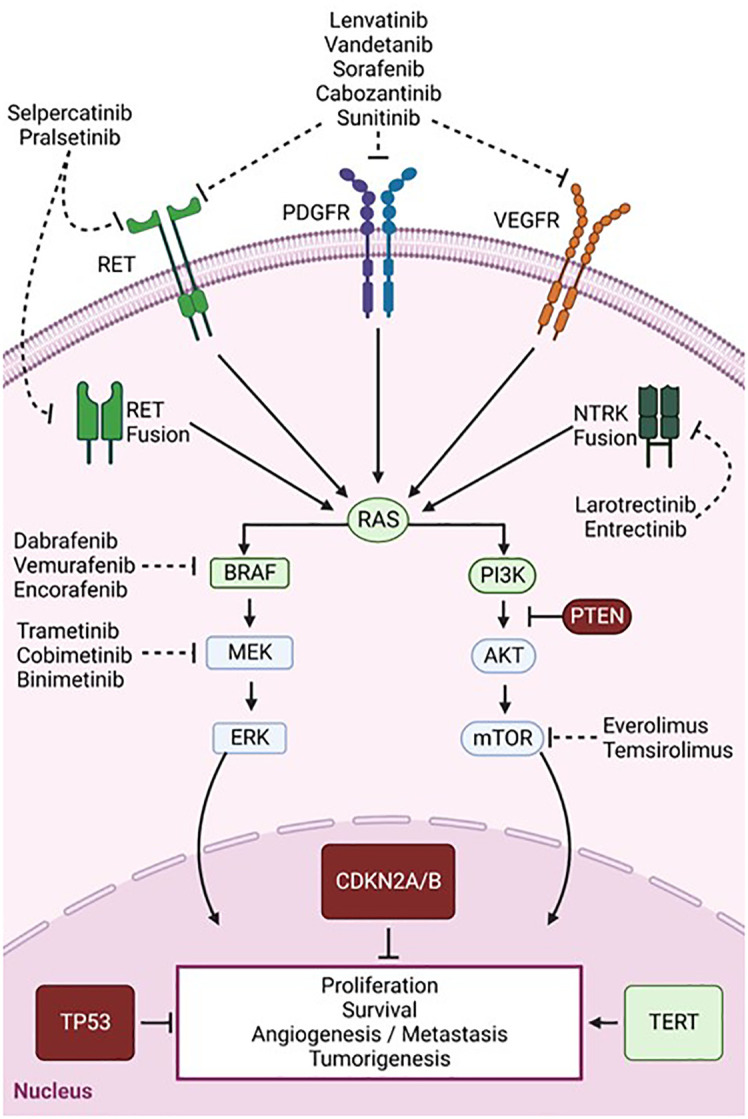
Signaling pathways and key mutations involved in thyroid cancer tumorigenesis and targeted therapies. Overview of the MAPK (left) and PI3K/AKT (right) pathways which are aberrantly activated in TC. Common mutations resulting in a loss or gain of function are depicted in red and green, respectively. Dashed arrows show targets of multi-kinase inhibitors (RET, PDGFR, and VEGFR) and single kinase inhibitors targeting BRAF, MEK, or mTOR. Created with BioRender.com.

During the last 10 years, major advances have been made in genomic profiling of TCs, which has uncovered some fundamental mutational schemes driving pathogenesis. Though TCs tend to have lower mutational burdens than other tumors such as lung cancer and melanoma, the mutational profiles heavily drive clinicopathological course and treatment strategies (59) (**Figure 1**). The main pathways that are highly implicated in thyroid tumorigenesis are the mitogen-activated protein kinase (MAPK) and phosphatidylinositol-3 kinase (PI3K)/AKT signaling cascades (**Figure 2**) (60). RAS, RAF, MEK, and ERK are the main protagonists of the MAPK pathway, which is involved in cell differentiation, proliferation, and apoptosis. Signal transduction through the MAPK pathway occurs after extracellular growth factors bind to a variety of receptor tyrosine kinases (RTKs) which lead to RAS activation and binding to BRAF, that subsequently leads to activation of MEK and ERK. TCs often harbor mutually exclusive mutations in the BRAF (such as BRAFV600E) or RAS (HRAS, NRAS, and KRAS isoforms) components of the MAPK pathway (60, 61).

The PI3K/AKT pathway is the second most frequently dysregulated pathway in TC. Although RAS is a classical dual activator of both PI3K/Akt and MAPK signaling, RAS mutations seem to preferentially activate the PI3K-AKT-mTOR pathway which is involved in cell proliferation and survival (62). Point mutations in the phosphatidylinositol-4,5-bisphosphate 3-kinase catalytic subunit alpha (PIK3CA) and phosphatase and tensin homolog (PTEN), a tumor suppressor and PI3K antagonist, also lead to PIK3/AKT pathway activation and promote thyroid tumorigenesis (60). Other common TC mutations activating MAPK and PI3K pathways include gene fusions of proto-oncogenes, such as those occurring in the rearrangement during transfection (RET) and neurotrophic-tropomyosin receptor kinase (NTRK) genes, which encode RTKs (63). Various mutations in genes involved in transduction and regulation of these pathways lead to constitutive activation of MAPK and PI3K/Akt signaling and ultimately to uncontrolled cell survival and proliferation (64).

Furthermore, genomic mutations have been found to be correlated to responses to RAI treatment. Overactivation of the MAPK pathway suppresses the expression of thyroid-specific genes required for iodine uptake such as the sodium iodide symporter, leading to RAI refractoriness (65, 66). Inhibition of BRAF or MEK has been shown to reverse this effect and restore RAI avidity (65). Further, exceptional responders to RAI were found to have an enrichment of RAS, class 2 BRAF, or RTK fusion mutations, which act through RAF dimerization, leading to a low MAPK transcriptional output. On the other hand, non-responders were associated with the BRAFV600E mutation, which signals as a monomer that is unresponsive to negative feedback, resulting in high flux through the MAPK pathway. They were also found to be enriched in mutations of genes regulating mRNA splicing and the PI3K pathway (67).

**PTC** has a relatively low mutational burden compared to other carcinomas, likely contributing to its slow growth and less aggressive clinical nature (55, 68). Alterations in BRAF, mainly BRAFV600E (61.7%), RAS (12.9%), and RET fusions (5%) are hallmark drivers in PTC (64). Based on a BRAFV600E-RAS gene expression score, PTCs may be grouped according to their molecular differences as BRAFV600E-like and RAS-like PTC. BRAFV600E mutation is linked to enhanced MAPK activation and is more frequent in classic and tall-cell variant PTC (64). Several studies have reported an association between the V600E variant and aggressive disease features such as RAI refractoriness, lymph node metastases, locoregional invasion, and recurrence (64, 67, 69). Interestingly, PTCs with coexisting mutations in BRAF and the telomerase reverse transcriptase (TERT) promoter are associated with aggressive clinicopathological characteristics, more so than either mutation alone (70, 71). On the other hand, RAS mutations occur mostly in follicular-variant PTC and non-invasive follicular thyroid neoplasm, which have a genomic profile more similar to FTC. RAS-like PTCs are associated with a decreased risk of recurrence, RAI uptake, and less aggressive phenotypes (64, 67, 69). Aside from alterations of the MAPK pathway, some well-differentiated PTCs have been reported to harbor mutations in EIF1AX as well as fusions within PPAR-γ, NTRK1/3, and THADA (72). Genomic analyses reveal that PTC bears a relatively stable genome, which could explain the usually indolent course of this disease. Nonetheless, transformation of PTC to ATC may occur and, therefore, continued study is necessary for identification of those PTCs early that will dedifferentiate and become aggressive and life-threatening.

In FTC, the most common mutations are in the RAS gene family (HRAS, KRAS, and NRAS), especially in the NRAS isoform, which has been found to be mutated in as many as 57% of RAS-mutant FTC cases (2). Although RAS mutations were proposed to be negative prognostic markers, they do not appear to be predictors of disease-specific mortality (73). Another standout genetic alteration in FTC is the PAX8-PPARγ gene rearrangement, which has been reported in multiple studies to occur at differing incidences (12-53%) but appears to have little correlation with survival, invasiveness, or prognosis (2, 74). TERT promoter mutations have been described in about 15% of FTCs and are associated with worse clinical and prognostic features (75). In fact, in a genomic analysis of advanced DTCs and ATC, TERT mutations were more commonly reported in widely invasive FTCs (91.67%) than any other subtype (75). Furthermore, point mutations of driver genes EIF1AX and DICER1 as well as somatic arm-level copy changes (loss of 22q) have been described in FTC, although their clinical significance still needs to be clarified (69). In FTC the total mutational burden has been reported to be a positively correlated predictor of mortality and recurrence, independent of histopathological classification (76).

Uniquely, OCAs harbor numerous mutations affecting the mitochondrial DNA (mtDNA) (71%), which is likely linked to the abundant mitochondrial load characterizing these cells. Sixty-seven percent of the mtDNA mutations observed occur in the genes encoding complex I of the electron transport chain. However, no significant correlation was observed between mitochondrial mutations and tumor aggressiveness, implicating mtDNA mutations in the tumorigenesis rather than progression of OCA (77). Like other DTCs, OCAs have significant dysfunction in the MAPK and PI3K/AKT/mTOR pathways caused by numerous somatic mutations in their components. Interestingly, overexpression of BRAF (12%) has been detected due to whole chromosome duplication of chromosome 7 in OCA tumors (77). At least one RTK was found to be mutated in 20% of OCAs such as RET (4%), MET (4%), EGFR (2%), and PDGFR (2%) (77). RAS mutations (15%) are also common with NRAS (9%) being the most commonly mutated isoform (77). Along with that, EIF1AX, NF1, TP53, CDKN1A mutations were detected in 11%, 9%, 7%, and 4% of OCAs, respectively (77). Further, they have also been reported to harbor mutations in TERT (22%), which are more common in the widely invasive (32%) than the minimally invasive subtype (5%) (77).

PDTC and DHGTC harbor driver mutations in BRAF and RAS. As DHGTC derives from PTC, it is more associated with BRAFV600E mutations (53%) and RAI refractoriness (54). On the other hand, PDTC more commonly harbors RAS mutations (48%) and has a higher rate of RAI avidity (54). As seen in other TCs, the driver mutation of either subtype is predictive of clinical behavior. RAS mutations are more correlated with enhanced tumor growth and risk of distant metastases while BRAF-mutants tend to be smaller and more prone to nodal metastases than distant disease (54, 68). Aside from BRAF/RAS mutations, high grade non-anaplastic TCs acquire additional genomic alterations which are responsible for their dedifferentiation. For instance, TERT mutations, which are known to be associated with more aggressive tumor behavior, were reported in 59% and 52% of DHGTCs and PDTCs, respectively (54). Although TP53, PTEN, and EIF1AX mutations were detected in both types, PDTC had significant enrichment in these mutations (54). Additionally, TERT, TP53 and PTEN mutations were associated with decreased distant-metastasis free survival (54). Gene fusions such as those involving RET, PAX8-PPARγ, ALK, and NTRK were detected in only 10% of either DHGTC or PDTC.

Similar to PTC and FTC, ATC has driver mutations in BRAF (19-45%) and RAS (9.5-27%); however, their frequencies are lower than in DTC (55, 68, 75). Due to the availability of effective targeted therapy, ATCs harboring BRAFV600E mutations have been reported to be associated with significantly enhanced overall survival (OS) compared to RAS-mutated ATC. On the other hand, tumors that are wild-type for both BRAF and RAS mutations have been found to be enriched in NF1 mutations and carry an intermediate OS (78). The two most frequent mutations occurring in ATC are TP53 and TERT promoter mutations, both reported to occur in about 65-73% of cases (55, 68). Interestingly, both mutations often coexist with BRAF and RAS mutations (78). Also, activating mutations in the PI3K/AKT pathway such as PTEN and PI3KCA occur more frequently in ATC (37%) than DTC (18%) (55). Further, ATC harbors mutations less typical for thyroid tumors in genes associated with the SW1/SNF chromatin remodeling complex (18%-36%), histone modification (19%), cell cycle regulation such as CDKN2A, CDKN2B, and CCNE1 (29%), and tumor immune regulation (PDL1, PDL2, and JAK2) (55, 68). Additionally, some fusions involving RET, ALK, and NTRK 1-3 genes have been reported at low incidences in ATC (63). Interestingly, in this panorama of mutations, four distinct subtypes of molecular patterns of ATC have been proposed: (1) type 1 ATC, BRAF-positive ATC, likely evolving from PTC; (2) type 2 ATC, NRAS-positive ATC, which may originate from FTC; (3) type 3 ATC, which carries RAS mutations or more atypical ones (PTEN, NF1 and RB1) and is likely to originate from FTC or from OCA; and (4) mixed ATC, which harbor loss-of-function mutations in the genes of cell-cycle regulators, such as CDKN2A and CDKN2B, and do not seem to derive from a pre-existing DTC (55, 68, 75, 79).

## Thyroid cancer cellular microenvironment

**Figure 3:**
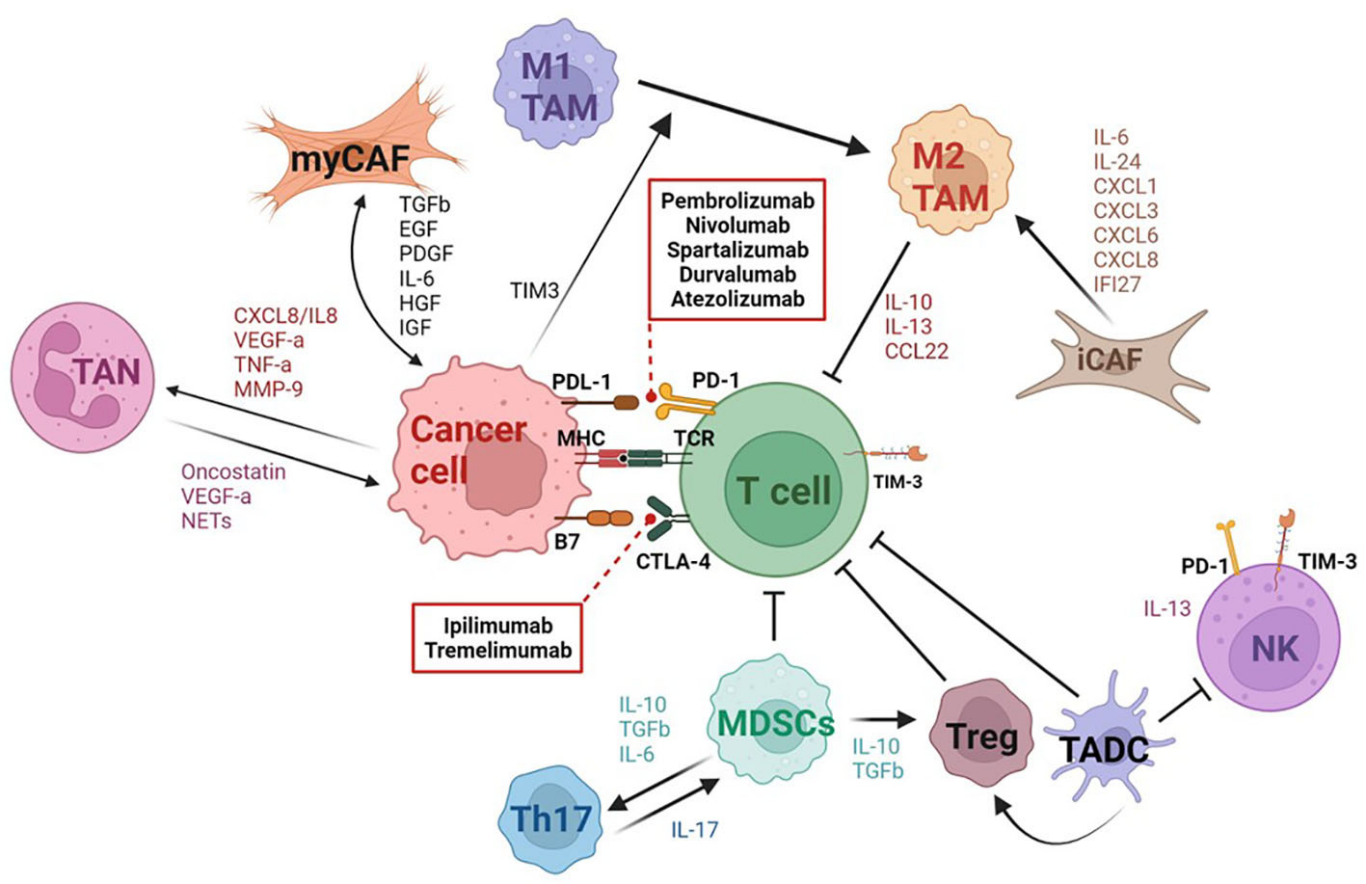
Schematic representation of the tumor microenvironment in thyroid cancer. Thyroid cancer (TC) is characterized by a complex tumor microenvironment (TME) with multiple interactions between tumor cells and various immune and stromal cells. Tumor cells induce activation and differentiation of fibroblasts into myCAFs or iCAFs by releasing multiple factors such as TGFβ, EGF, PDGF, HGF, IGF, etc. In return, myCAFs promote tumor progression and angiogenesis. The iCAF subset attracts and induces suppressive functions of myeloid cells by releasing inflammatory cytokines. ATC tumor cells induce M2 macrophage polarization through TIM3 expression. M2 macrophages and MDSCs play a key role in inhibiting T cell effector function in TC. Immature DCs also suppress the cytolytic functions of T cells and CD56^dim^CD16^+^ NK cells in ATC. Tumor cells also recruit neutrophils which act to promote cancer cell proliferation and invasiveness. The TME in ATC is characterized by an expansion of exhausted CD8^+^ T cells expressing PD1, CTLA4, and TIM3 and of an immunosuppressive NK subset (CD56^bright^ CD16^low^). Dashed arrows show immune checkpoint inhibitors targeting PD-1 and CTLA-4. Created with BioRender.

Understanding interactions between tumor cells and other components of the TME is crucial to effectively direct immunotherapeutic approaches in the treatment of TC, particularly for those not responsive to conventional therapies. In TCs, the TME is composed of cancer associated fibroblasts (CAFs) and various immune cells categorized as tumor associated myeloid (TAMC) or lymphoid (TALC) cells (80). TAMCs include macrophages (TAMs), myeloid-derived suppressor cells (MDSCs), neutrophils (TANs), and dendritic cells (TADCs), while TALCs include T cells and NK cells (69). Based on recent genomic analyses, in contrast to DTC and PDTC, ATC has higher number of immune infiltrates in TME, particularly of type 2 TAMs and dysfunctional/exhausted T cells and NK cells (81–83). In addition, ATC is characterized by high expression of PD-L1 compared to other TC subtypes (16). Based on this altered immune profile, ATC tumors may potentially benefit from ICI therapy, adoptive T and NK cell therapies as well as therapeutic strategies targeting TAM populations. Moreover, due to ATC’s relatively high tumor burden, neoantigen vaccines also offer a promising therapeutic scheme. For each TC subtype, the TME composition is unique with distinct interplay between the immune, stromal, and tumor cells. Understanding these interactions not only provides multiple targets for therapies but also allows for personalized approaches to potentially enhance outcomes for patients. Here, we detail the different cell subsets involved in conferring the pro-tumorigenic nature of the TME in ATC and other TCs (**Figure 3**).

Cancer-Associated Fibroblasts (CAFs) are involved in the development and progression of TC through cell proliferation and extracellular matrix (ECM) remodeling (84). CAFs are derived from various sources including resident tissue fibroblasts, mesenchymal stem cells, bone-marrow derived fibroblasts, adipocytes, endothelial, and epithelial cells and are activated in response to the secretion of soluble factors such as TGF-β, epidermal growth factor (EGF), reactive oxygen species (ROS), platelet-derived growth factor (PDGF), and interleukin (IL)-6 from thyroid tumor cells (85). In turn, CAFs promote cancer cell growth, invasion, angiogenesis, and metastasis through production of various growth factors (vascular endothelial growth factor (VEGF), EGF, connective tissue growth factor (CTGF), insulin-like growth factor (IGF), and hepatocyte growth factor (HGF)), cytokines (IL-6, IL-11, and IL-17), chemokines (CCL7, CCL5, CXCL12, and CXCL7), and the extracellular matrix (ECM) molecules (collagen, fibronectin, elastin, laminin) (84, 85). CAFs are recruited in the stroma at the tumor invasive front where they synthesize and deposit collagen, which is then cross-linked by the thyroid tumor-cell derived enzyme LOX. Collectively, this coordinated action leads to matrix stiffness and progression from PTC to the less differentiated PDTC and ultimately ATC (86).

CAFs are classified into two major subsets: myofibroblasts (myoCAF), highly expressing genes such as ACTA2, MCAM, MYH11, and TAGLN, and inflammatory fibroblasts (iCAFs), which overexpress genes involved in inflammation regulation such as CXCL1, CXCL6, CXCL8, IL-32, C1S, and C1R (83). The ATC-derived CAFs are mostly iCAFs, whereas the PTC-derived CAFs are mostly myoCAFs (83). In addition, ATC-derived CAFs are characterized by high expression of cytokines and chemokines, including CXCL1, CXCL3, CXCL6, CXCL8, IL6, IL-24, and IFI27 (83). Thyroid CAFs promote TC growth and progression by increasing the expression of immune checkpoints such as CTLA4, PDL1/2 and IDO1 and downregulation of CD8^+^ T cells and endothelial cells (87). Patients with a high CAF score had remarkably increased risk of aggressive outcomes in both ATC and PTC. Additionally, a high CAF score in TC patients was shown to be positively correlated with an increased expression of immune checkpoint markers, such as PD-L1, PD-L2, CD86, CD80 and CTLA4, and an increased expression of markers of activated TAMs, including EMR1, CSF1R, CD163 and ITGM in ATC and PTCs (88). Consequently, further studies are required to identify molecular signaling pathways regulating the immune modulating role of CAFs in order to design potential novel therapeutic approaches able to abolish the pro-tumorigenic immunity seen in TC.

### Tumor infiltrating immune cells

Tumor-associated macrophages (TAMs) are the largest component of infiltrating immune cells, representing more than 50% of the total cells, and are generally associated with poor survival in TC (15, 89, 90). They are subdivided into the pro-inflammatory/anti-tumor M1 (CD64, IDO, SOCS1, CXCL10, TNF-α and IL-1) and the anti-inflammatory/pro-tumor M2 (MRC1, TGM2, CD23, CCL22, IL-10 and IL-13) macrophages. While TAMs compose a smaller percentage of the total cells in PTC, they tend to be positively associated with more aggressive pathologies such as larger tumor sizes and lymph node metastasis (91). Compared to PTCs, the ATC TME is characterized by a polarization toward M2 macrophages (SELENOP+, SPP1+MARCO+, and SPP1+TGFBI+) and a decrease in M1 macrophages (IL-1B+, FCGBP+, and TXNIP+) (68, 83, 92–94). Soluble factors produced by ATC cells induce pro-tumor M2-like polarization of monocytes through T-cell immunoglobulin and mucin-domain containing protein-3 (TIM3). TIM3 and CSF1R expression as well as several pathways, such as E2F targets, IL-6-JAK-STAT3, and G2M checkpoint are positively correlated with the TAM-related prognostic index and T cell dysfunction in ATC (94). FZD6, RBBP8, PREX1 and HSD3B7 expressed by M2 macrophages are prognostic factors that are correlated with proliferation and invasion of ATC (95, 96). Recently, IL2RA+VSIG4+ TAMs have been identified as an ATC-specific subset with bifunctional M1 and M2 phenotype signatures that correlate with high lymphocyte infiltration and better prognosis (79). TCs could benefit from therapies that will deplete M2 TAMs or reprogram M2 towards an M1 phenotype; thus, further preclinical research and clinical trials need to be conducted to assess its potential application.

Myeloid derived suppressor cells (MDSCs) are a subtype of myeloid cells known to have an immunosuppressive function in cancer through ROS, arg-1, nitric oxide (NO), IL-10, TGFβ, cyclooxygenase 2 (COX-2) and PD-L1 and are usually associated with a poor prognosis (97). Peroxynitrite (PNT), the product of the interaction between superoxide and NO, could cause nitration of T cell receptor-CD8 complex, reducing its binding to the peptide MHC class I complex and rendering T cells unresponsive to antigen-specific stimulation (98). PNT has also been shown to hamper the recognition of cancer cells by cytotoxic T lymphocytes (98). Further, accelerated depletion of L-arginine and cysteine in the TME caused by MDSCs results in decreased CD3 chain expression, diminished production of IL-2 and IFN-γ, and inhibited T cell proliferation (98). MDSCs have also been shown to alter immune activity by promoting Foxp3^+^ T regulatory (Treg) cell differentiation via IL-10 and TGFβ secretion as well as enhancing Treg trafficking to tumor sites through CCR5 (98). MDSCs can also activate Th17 cells by secretion of IL-6 and TGF-β (99). IL-17 increased the immuno-suppressive function of MDSCs through the upregulation of arg-1, matrix metalloproteinase 9 (MMP-9), indoleamine 2,3-dioxygenase (IDO), and COX-2 (99). Circulating MDSCs are significantly higher in ATC patients compared to healthy controls and correlated with increased serum levels of IL-10 (97). Along with that, long-term survivor (LTS) ATC patients, who have survived longer than two years, display lower numbers of tumor-infiltrating MDSCs compared to ATC control patients (100).

Tumor-associated dendritic cells (TADCs) display an immature phenotype characterized by low levels of co-stimulatory molecules, high levels of inhibitory molecules and the production of immunosuppressive cytokines (IL-10 and TGF-β), which lead to poor T and NK cell-mediated immune responses (101, 102). Further, it has been suggested that TADCs also contribute to tumorigenesis through crosstalk with Tregs (101). Interruption of the DC and Treg axis could be a promising therapeutic strategy to quell the immunosuppressive TME in TC. The function of TADCs can be restored by blocking immunosuppressive pathways, such as those associated with PD-1, IL-10 secretion, and lactic acid production (101). While TADCs infiltration has been reported in PTCs, further studies are required to determine DC involvement in ATC (103, 104). To combat the immunosuppressive effects of TADCs, neoantigen-based DC vaccine therapy has been explored as a treatment option in TC. A phase I clinical study demonstrated that mature DC vaccination combined with low-dose IL-2 was well tolerated when administered to advanced PTC and FTC patients (105). Presently, there is not a recognized ATC-specific antigen. However, as ATC has a relatively high mutation burden, the application of a DC vaccine has potential as a treatment in ATC, with need for further exploration and development of tumor-specific antigens and neoantigens.

Tumor-associated neutrophils (TANs) have a controversial role in cancer despite their inflammatory function. On one hand, TANs are able to kill tumor cells, stimulate the T cell-dependent anti-tumor response, and inhibit angiogenesis (106–108). On the other hand, TANs can favor genetic instability in cancer cells and release cytokines (oncostatin-M, VEGF-A) or granule proteins (neutrophil elastase) that are involved in the promotion of cancer cell proliferation, invasiveness, and angiogenesis (109–111). In fact, for TCs, the neutrophil-to-lymphocyte ratio (NLR) has been explored as a prognostic indicator, in which a high NLR has been associated with aggressive forms of TCs such as PDTC and ATC and/or poor treatment responses (112, 113). TC-cell released factors such as CXCL8/IL-8 and GM-CSF recruit neutrophils and significantly improve their survival. Furthermore, TCs induced the production of factors by TANs (ROS, the expression of proinflammatory and angiogenic mediators (CXCL8/IL-8, VEGF-A, and TNF-α), and the release of MMP-9) that can retain the ability to promote tumor progression. ATCs induce neutrophil extracellular DNA trap (NET) release, whereas PTCs or normal thyroid cells did not. ATCs-induced NET production occurred in a ROS-dependent and cell death-independent manner and was associated with mitochondrial reactive oxygen species production, mitochondrial DNA release, and ATC cell growth (114). Further research is needed to understand the mechanisms by which neutrophils influence TC development and progression.

Natural killer (NK) cells are known to have an anti-tumor function by directly killing tumor cells via granzyme B and perforins (115). Two NK subgroups have been identified: 1) CD56^dim^CD16^+^ NK cells with typical cytotoxic functions and 2) CD56^bright^CD16^-/low^ NK cells which are only weakly cytotoxic and have a more immunoregulatory role, mediated through the secretion of IL-13 (116, 117). Compared to healthy individuals, patients with PTC have a significant enrichment of the dysfunctional CD56^bright^CD16^-/low^ NK cells (117). Further, ATC patients have been characterized by an increased frequency of the CD56^hi^CD16^hi/lo^ NK subset with significantly reduced cytotoxicity and high expression of exhaustion markers such as PD-1 and TIM3 (14, 115). PD-1 and TIM3 blockade reinvigorated cytotoxicity of both the dysfunctional CD56^hi^CD16^hi/lo^ and the more functional CD56^lo^CD16^hi^ NK cell subsets from ATC patients, suggesting that NK cells might be potential treatment targets in advanced thyroid cancers (115). Moreover, patients with ATC may benefit from NK cell-based immunotherapy as ATC tumor-derived NK cells display a suppressed phenotype due to downregulated expression of natural killer group 2, member D (NKG2D), a constitutively expressed NK cell receptor which is critical for cancer immunosurveillance (118). In fact, in a preclinical pulmonary metastasis model of ATC, NK cells were able to target metastatic ATC; highlighting that NK cell-based immunotherapy may serve as an effective therapeutic approach for ATC (82).

Tumor-infiltrating T cells are the heterogeneous population that include both the antitumoral CD8^+^ cytotoxic T cells (CTLs) and T helper (Th1) cells and the pro-tumoral Th2 and Treg cells (119–123). In PTC patients, the CD8^+^ CTL, CD4^+^ T cells and B cell infiltration is associated with better outcomes and enhanced survival rate (124). Tregs are involved in the suppression of immune responses, favoring disease progression and lymph node metastases in various cancers (14). A high infiltration of Treg has been reported in PTC tumors and metastatic lymph node tissues when compared to multinodular goiter patients and it was associated with the aggressiveness and recurrence of the PTC (124). Moreover, CD4^+^ and CD8^+^ T cells displayed functional exhaustion in patients with metastatic DTC (125, 126). While ATC displays enhanced immune infiltration compared to PTC, TILs characterized by expression of T-cell exhaustion markers such as cytotoxic T-lymphocyte-associated protein 4 (CTLA-4), PDL-1/PDL-2, PD-1, PVR, TIGIT, LAG3, and TIM-3 (14). The role of other major CD4^+^ T cell subsets such as Th17 cells and follicular helper T (Tfh) cells in TC have not been studied thoroughly (14). Moreover, follicular CD8^+^ T cells (CD8^+^CXCR5^+^) are significantly increased in ATC tumors compared to healthy PBMCs; however, their function is still unclear (127). Collectively, enhancement of exhausted T cells in ATC warrants clinical trials of immune-based cancer therapy including immune checkpoint inhibitors, adoptive T cell, and CAR-T (ICAM-1 CAR-T) cell therapies.

## Current treatments and future directions

Neck surgery and selected use of RAI have been the mainstay of therapy in differentiated thyroid cancers for many years. However, historically, therapeutic options for patients with metastatic disease refractory to RAI were limited. In the past decade, treatment of advanced DTCs has undergone major advancements. Broader access to next generation sequencing of tumors and better understanding of tumor biology has opened the horizons to novel targeted therapies which have led to significant improvements in the prognosis of these patients. The molecular profile of tumors, disease burden and rate of progression, as well as patient comorbidities should all be taken into account when considering the optimal drug and timing for initiation of systemic therapy, as will be discussed in the following sections.

### Differentiated thyroid cancer

The vast majority of DTCs can be treated with surgery, followed by RAI in selected cases. The decision regarding type of surgery is based on the extent of disease, presence of lymph node metastases, as well as the patient’s comorbidities and preference (128). Lobectomy should be considered for small tumors measuring less than 4 cm with no evidence of gross extrathyroidal extension. When carefully selected, patients undergoing a lobectomy have comparable overall survival as those treated with a total thyroidectomy (129, 130). Additionally, with adequate follow-up, the rare cases of disease recurrence after an initial lobectomy can be readily detected and treated. Following total thyroidectomy, RAI should be considered in patients at intermediate or high risk of disease recurrence per the American Thyroid Association (ATA) risk classification (128). TSH suppression should also be considered, based on recurrence risk (128).

Although DTC has an excellent prognosis, a minority of patients will develop distant metastases, most frequently to the lungs and bones (131). As long as these metastases remain RAI-avid, prognosis remains favorable (132). However, half of metastatic DTCs become refractory to RAI, which is associated with a poor prognosis and a 10-year overall survival of barely 10% (133). Yet, given the indolent, slowly progressive course of disease in most of these patients, they can be initially watched under TSH suppression alone, with regular imaging, laboratory workups and clinical follow-ups (7, 72, 128). Locoregional therapy to oligo-progressive disease, such as stereotactic radiation or surgery, can be considered during this observation period (7, 72, 131, 132, 134). If localized therapy is not feasible, disease becomes symptomatic, and/or there is significant progression in multiple sites of disease, then initiation of systemic therapy becomes warranted (7, 72, 131, 132, 134). As genetic-informed targeted therapy is FDA approved for TCs with certain mutations or fusions, it is important to obtain molecular profiling of the tumor when faced with a patient with metastatic DTC needing systemic therapy.

There are multiple drugs or drug combinations that are approved for DTC, of which 5 require a particular mutation or fusion to be present in the tumor (**Table 1**). The remaining drugs are all anti-angiogenics which do not require this information. The optimal sequencing of anti-angiogenics and genetic-informed therapies is currently an area of debate.

**Table 1:** Summary of clinical trials for kinase inhibitors in differentiated and anaplastic thyroid cancers.

Thyroid cancer subtype	Drug	Target	Number of subjects	Study population	Efficacy results	Reference
**DTC**	**Cabozantinib**	VEGFR2, AXL, MET, RET, C-KIT	170	RR-DTC with prior progression on sorafenib and/or lenvatinib	ORR: 11% Median PFS: 11.0 months	Brose et al. (20)
	**Dabrafenib** **(Single agent)**	BRAF V600E	26	BRAF V600E-mutated RR-DTC with progressive disease	ORR: 35% Median PFS: 10.7 months	Busaidy et al. (21)
	**Dabrafenib + trametinib**	Dabrafenib: BRAF V600E Trametinib: MEK	27	BRAF V600E-mutated RR-DTC with progressive disease	ORR: 30% Median PFS: 15.1 months	Busaidy et al. (21)
	**Entrectinib**	NTRK fusions, ALK, ROS1	13	NTRK fusion-positive TC with locally advanced or metastatic disease	ORR: 53.8% Median PFS: 19.9 months	Demetri et al. (22)
	**Larotrectinib**	NTRK fusions	22	NTRK fusion-positive TC with locally advanced or metastatic disease	ORR: 71% 24-month PFS: 84%	Waguespack et al. (23)
	**Lenvatinib**	VEGFR1-3, RET, FGFR1-4, PDGFR, KIT	261	RR-DTC with progressive disease	ORR: 64.8% Median PFS: 18.3 months	Schlumberger et al. (24)
	**Pralsetinib**	RET fusions and mutations	21	Previously treated RET fusion-positive TC with locally advanced or metastatic disease	ORR: 84% Median PFS: 25.4 months	Subbiah at al (25)
	**Selpercatinib**	RET fusions and mutations	19	Previously treated RET fusion-positive TC with advanced or metastatic disease and indication for systemic therapy	ORR: 79% Median PFS: 20.1 months	Wirth et al. (26)
	**Sorafenib**	VEGFR1-3, RET, RAF, PDGFR-β	207	Locally advanced or metastatic RR-DTC with progressive disease	ORR: 12.2% Median PFS: 10.8 months	Brose et al. (27)
**ATC**	**Dabrafenib + trametinib**	Dabrafenib: BRAF V600E Trametinib: MEK	36	Unresectable or metastatic BRAF V600E-mutated ATC	ORR: 56% Median PFS: 6.7 months	Subbiah et al. (28)
	**Lenvatinib**	VEGFR1-3, RET, FGFR1-4, PDGFR, KIT	34	TKI-naïve ATC (regardless of mutation)	ORR 2.9% Median PFS: 2.6 months	Wirth et al. (29)
	**Sorafenib**	VEGFR1-3, RET, RAF, PDGFR-β	20	ATC which previously progressed on ≤ 2 lines of cytotoxic chemotherapy not amenable to curative surgery or radiation	ORR: 10% Median PFS: 1.9 months	Savvides et al. (30)

DTC, differentiated thyroid cancer; ATC, anaplastic thyroid cancer; VEGFR1-3, VEGF receptors 1-3; FGFR1-4, FGF receptors 1-4; RR-DTC, radioiodine refractory DTC; TC, thyroid cancer; TKI, tyrosine kinase inhibitor; ORR, overall response rate; PFS, progression-free survival.

The multikinase-inhibitors (MKIs) sorafenib and lenvatinib are approved as a first-line therapy for locally advanced or metastatic RAI-refractory DTC and PDTC. These drugs are potent inhibitors of the vascular endothelial growth factor receptors (VEGF-R) 1 -3 and have variable inhibitory action on other tyrosine kinases, including the fibroblast growth factor (FGF) and platelet-derived growth factor (PDGF) receptors. In the phase III randomized, double-blind, placebo-controlled DECISION trial, sorafenib led to a significant 5-month prolongation of progression-free survival (PFS) compared to placebo (10.8 vs 5.8 months) in patients with progressive RAI-refractory DTC (27). The following year, the SELECT phase III double-blind, placebo-controlled trial of lenvatinib in a similar patient population was published. In this study, median PFS with lenvatinib was significantly longer than in the placebo group, at 18.3 months versus 3.6 months (24). Given this significant prolongation in PFS, lenvatinib has become the treatment of choice in advanced RAI-refractory DTC with no actionable mutation, since its FDA approval in 2015. More recently, in 2021, cabozantinib, another MKI, was approved as a second-line therapy for patients with locally advanced or metastatic RAI-refractory DTC that have progressed on prior antiangiogenic therapy. Cabozantinib inhibits the VEGF-R, but also has activity against other tyrosine kinases involved in tumor growth and angiogenesis including AXL and c-MET, which have been implicated in resistance to antiangiogenics (135, 136). The COSMIC-311 trial, which led to the FDA approval of cabozantinib, is a double-blind phase III placebo-controlled trial in patients with RAI-refractory DTC who have progressed on ≤ 2 prior anti-VEGF-R MKIs. This trial showed significant prolongation of PFS with cabozantinib, with a median of 11.0 months compared to 1.9 months with placebo (20). Nevertheless, despite their efficacy, MKIs are associated with significant toxicity related to their VEGF-R inhibition, including hypertension, palmar-plantar erythrodysaesthesia syndrome, stomatitis and weight loss.

As previously mentioned, BRAFV600E is the most frequent driver mutation in PTCs. The BRAF inhibitor dabrafenib with or without the MEK inhibitor trametinib, showed encouraging overall survival (OS) and PFS outcomes in metastatic BRAFV600E-mutated PTC and should be considered in these patients (21). Moreover, although less frequent, thyroid cancers can be driven by chromosomal rearrangements, including RET, NTRK, ALK and BRAF fusions (137). Specific kinase inhibitors targeting each of these fusions are available and have shown notable efficacy in TC. The LIBRETTO-001 trial looking at the RET-inhibitor selpercatinib, which included 19 patients with RET fusion-positive DTC previously treated with at least one systemic therapy, showed an overall response rate (ORR) of 79% and a median PFS of 20.1 months (26). Similarly, the selective RET-inhibitor pralsetinib showed an ORR of 84% and a median PFS of 25.4 months in previously treated RET fusion-positive DTC (25). In NTRK fusion-positive DTC, the selective TRK inhibitors larotrectinib and entrectinib have also shown prolonged OS and PFS, with a 24-month PFS of 84% with larotrectinib and a median PFS of 19.9 months with entrectinib (22, 23). Moreover, selective ALK inhibitors have been successfully used in case reports of ALK fusion-positive DTCs (138, 139). Due to minimal off-target activity, these selective kinase inhibitors have more acceptable toxicity profiles compared to antiangiogenic kinase inhibitors, justifying their choice as first-line therapies when possible.

Another therapeutic avenue that is being increasingly recognized for RAI-refractory advanced TC is redifferentiation therapy. This strategy aims to restore RAI uptake through inhibition of MAPK signaling. In fact, increased MAPK pathway activation, such as in the presence of a BRAFV600E mutation, leads to decreased NIS expression and tumor dedifferentiation, ultimately rendering RAI ineffective (140). Several studies have shown that by inhibiting MAPK signaling with mutation-specific kinase inhibitors, RAI uptake can be restored, allowing subsequent I^131^ therapy in a tumor which was previously non-RAI avid. Redifferentiation therapy has been attempted using MEK inhibitors in RAS-mutant tumors (141–145), BRAF ± MEK inhibitors in BRAFV600E-mutant tumors (142, 143, 146–149), and even NTRK or RET inhibitors in patients harboring corresponding fusions (150–153). Data thus far show promising results with this strategy, allowing disease control in many patients and potentially delaying the need for systemic therapy with kinase inhibitors. For instance, in the recently published prospective multicentric phase II MERAIODE trial, Leboulleux and colleagues treated patients with RAI-refractory progressive DTC with an empiric dose of 150 millicuries of RAI after a short course of kinase inhibitors. Patients with a BRAFV600E mutation were treated with dabrafenib + trametinib, while those with a RAS mutation were treated with trametinib alone. At 6 months, 90% of patients had either stable disease (18/31) or a partial response (10/31) in both BRAF and RAS mutated cohorts. Nevertheless, available trials are very heterogenous such that many questions remain unanswered, including ideal candidates for redifferentiation, duration of targeted therapy prior to RAI administration and optimal dose of RAI. Therefore, more studies are needed to further determine how to best use this approach, which patients are most likely to benefit from it and potential long-term risks (154). Some data suggest that RAS mutation, higher thyroglobulin levels, smaller tumor diameter and lower ^18^FDG uptake on PET/CT could predict success of redifferentiation therapy (149, 155). An ongoing phase 2 clinical trial investigating the efficacy of selpercatinib in restoring RAI uptake in RET fusion-positive RAI-refractory TC may help answer some of these questions (NCT05668962).

One major gap in the currently available therapies is related to the lack of specific kinase inhibitors targeting RAS, which is the second most frequent driver mutation in TC. BRAF inhibitors, which target the MAPK pathway downstream of RAS, are ineffective in RAS-mutant tumors because they lead to a paradoxical activation of MAPK signaling through dimerization of nonmutant RAF isoforms in the presence of active RAS (156–158). Thus, several new drug classes are currently under investigation for the treatment of RAS-altered tumors. These include pan-RAF and RAF dimer inhibitors, which have high biding potencies to all RAF isoforms, therefore overcoming the paradoxical MAPK activation that occurs with first generation BRAF inhibitors (157, 158). Multiple new RAF inhibitors have shown efficacy *in vitro* and are currently being investigated in phase I clinical trials: ERAS-254 (NCT05907304), DAY101 (NCT04985604), BGB-3245 (NCT04249843), KIN-2787 (NCT04913285), JZP815 (NCT05557045). Small molecules directly inhibiting ERK1/2, which target the MAPK pathway signaling downstream of both BRAF and RAS kinases, are also being studied in clinical trials, including BVD-523 (NCT04488003) and LY3214996 (NCT04534283) (159–161).

Pan-RAF, RAF dimer and ERK kinase inhibitors also represent potential therapeutic options for patients harboring class II/III BRAF alterations. While the class I BRAF V600x mutations allow BRAF to act as a constitutively active monomer, class II/III mutations signal through BRAF homo- or heterodimers (162). Yet, first generation RAF inhibitors selectively target BRAF monomers, making then ineffective against class II/III mutations, Pan-RAF kinase inhibitors suppress the activity of both monomeric and dimeric forms of BRAF and therefore can target all BRAF mutations and oncogenic fusions. Similarly, the mechanism of action of RAF dimer and ERK inhibitors make them also effective against class II/III BRAF mutations.

Although showing good initial responses to kinase inhibitors, both selective agents and MKIs, many patients with advanced DTC will eventually progress due to acquired resistance to therapy (7, 163). For instance, acquired secondary RAS mutations have been described as a mechanism of resistance to BRAF inhibitors (163). These could potentially respond to RAF or ERK inhibitors if these drugs were proven to be clinically effective in RAS-mutant TC. Paradoxical BRAF dimerization can also lead to resistance to BRAF inhibitors. Paradox-breaker BRAF inhibitors, such as PLX8394 and CFT1946, are potent, highly selective drugs that inhibit BRAF dimerization and do not lead to paradoxical pathway reactivation (164). These drugs have shown promising *in vitro* efficacy (165, 166). A phase I/II trial of CFT1946, a bifunctional degradation activating compound degrader, is currently underway in BRAFV600E-mutant solid tumors (NCT05668585). Next generation small molecule BRAF inhibitors, which offer a more potent BRAF blockade, are also under investigation in patients who failed first-generation drugs. Preliminary results from the phase I trial looking at the new BRAF inhibitor ABM-1310 in patients with BRAFV600E-mutated solid tumors showed favorable safety and efficacy, including in patients previously refractory to BRAF/MEK inhibitors (NCT04190628) (167). Finally, novel combinations with BRAF inhibitors are also being explored to overcome treatment resistance. Notably, Serum Glucocorticoid-Regulated Kinase 1 (SGK1) signaling has been found to maintain MAPK and PI3K activation in patients on BRAF + MEK inhibitors, leading to resistance to therapy. A novel SGK1 inhibitor (THRV-1257) has shown promising efficacy *in vitro* in ATC cell lines and will soon be investigated in a phase I clinical trial (168). Increased expression and activation of HER2/HER3 tyrosine kinase receptors was also suggested to play a role in resistance to BRAF inhibitors (169). Thus, the HER kinase inhibitor lapatinib is currently being investigated in combination with dabrafenib in refractory BRAFV600E/K mutated thyroid cancers (NCT01947023).

Immune checkpoint inhibition has also been studied in DTC as one of the potential strategies to delay progression or as a salvage therapy at progression (**Table 2**). As previously mentioned, some advanced DTCs have an immunosuppressive TME and high PD-L1 expression, making them suitable for immune checkpoint inhibition (126, 170, 171). However, results with single-agent immunotherapy in DTC have not been promising. In the KEYNOTE-028 phase Ib trial, 22 patients with PD-L1 positive, locally advanced or metastatic DTC, treated with single agent pembrolizumab for 24 months or until progression or unacceptable toxicity, had a with a median PFS of 7 months and an ORR of 9% (46). Combination of immunotherapy with kinase inhibitors was then explored. Addition of pembrolizumab to the MKI lenvatinib was studied in a phase II clinical trial (NCT02973997). Preliminary results reported in a poster at the 2020 American Society of Clinical Oncology (ASCO) meeting showed a 12-months PFS of 74%, which may be attributable to the lenvatinib alone (47). Another ongoing trial is looking at the combination of cabozantinib with the anti-PD-L1 atezolizumab in advanced solid tumors, including DTC (NCT03170960). Results in 31 patients with metastatic and/or progressive RAI-refractory DTC were presented in a highlighted poster at the 2022 ATA meeting, showing a promising median PFS of 15.2 months (44). Multiple other clinical trials of immunotherapy in advanced DTC are ongoing, including a phase II trial of the combination of the anti-CTLA4 ipilimumab with the anti-PD1 nivolumab (NCT03246958), a phase II trial of cabozantinib + ipilimumab/nivolumab (NCT03914300), a phase II trial of the BRAF inhibitor encorafenib + MEK inhibitor binimetinib with or without nivolumab in metastatic BRAFV600E-mutated RAI-refractory DTC (NCT04061980), and a phase II trial of the anti-PD-L1 durvalumab with the anti-CTLA4 tremelimumab (NCT03753919).

**Table 2:** Summary of available clinical data with immunotherapy in differentiated and anaplastic thyroid cancers.

Thyroid cancer subtype	Drug	Number of subjects	Study	Study population	Efficacy results	Reference
**DTC**	**Atezolizumab +** **cabozantinib**	31	COSMIC-021 (NCT03170960), Phase Ib	Treatment-naïve, locally advanced, metastatic and/or progressive RR-DTCs	ORR: 42% Median PFS: 15.2 months	Taylor et al. (44)
	**Ipilimumab/nivolumab + cabozantinib**	11	NCT03914300, Phase II	Locally advanced or metastatic RR-DTCs that have progressed on one previous anti-VEGFR therapy	**Interim results** ORR: 18% Median PFS: 9 months	Konda et al. (45)
	**Pembrolizumab**	22	KEYNOTE-028 (NCT02054806), Phase Ib	Locally advanced or metastatic DTC	ORR: 9% Median PFS: 7 months	Mehnert et al. (46)
	**Pembrolizumab + lenvatinib**	30	NCT02973997, Phase II	Treatment-naïve, RR-DTC with progression ≤ 14 months prior to enrollment	ORR: 62% 12-month PFS: 74%	Haugen et al. (47)
**ATC**	**Atezolizumab + vemurafenib/cobimetinib**	18	NCT03181100, Phase II	Locally advanced and/or metastatic ATC	ORR: 72% 24-month OS: 67%	Cabanillas et al. (48)
	**Durvalumab + tremelimumab + SBRT**	13	Phase I	Metastatic ATC incurable by surgery or radiation therapy	ORR: 0% Median OS: 104 days	Lee et al. (49)
	**Pembrolizumab + kinase inhibitor**	12	Retrospective cohort study	ATC patients in whom pembrolizumab was added at progression on a kinase inhibitor	ORR: 42% Median PFS from addition of pembrolizumab: 3.0 months	Iyer et al. (50)
	**Pembrolizumab or nivolumab**	13	Case series	Locally advanced and/or metastatic ATC treated with a PD-1 inhibitor	ORR: 16% Median PFS: 1.9 months	Hatashima et al. (51)
	**Pembrolizumab + lenvatinib**	35	ATELP (EudraCT No. 2017-004570-3), Phase II	Metastatic ATC	ORR: 51.9% Median PFS: 10 months	Dierks et al. (52)
	**Spartalizumab**	42	Phase II	Locally advanced and/or metastatic ATC	ORR: 19% Median PFS: 1.7 months	Capdevila et al. (53)

RR-DTC, radioiodine refractory differentiated thyroid cancer; ATC, anaplastic thyroid cancer; ORR, overall response rate; PFS, progression-free survival; OS, overall survival; SBRT, stereotactic body radiation therapy.

Beyond immune checkpoint inhibition, other immune-targeting therapies are under investigation in advanced TC, including chimeric antigen receptor modified T-cells (CAR-Ts). These are genetically engineered T-cells reprogramed to recognize and eliminate tumor cells expressing specific antigens (172). CAR-Ts have demonstrated remarkable efficacy in hematological malignancies but are more challenging to develop for solid tumors, due to more difficult tumor-specific antigen selection and an immunosuppressive TME that impedes access of CAR-Ts into the tumor (173). The TSH-receptor, a well-known thyroid specific antigen, seems to be a promising target for CAR-Ts in advanced DTCs in *in-vitro* and mouse models (174).

### Anaplastic thyroid cancer

While ATC was historically been known as a highly lethal malignancy with a median OS of only 5 months, kinase inhibitors and immunotherapy have revolutionized the management of this disease over the past few years (175) (**Tables 1**, **2**). Treatment of patients with ATC differs significantly from those with DTC. Given that ATC is a rapidly progressive malignancy which often presents with locoregional advanced disease and distant metastases, expedited initiation of the appropriate treatment is crucial. In patients with stage IVB disease, surgical resection of the primary tumor, when feasible, followed by high-dose external beam radiation therapy to the neck with concomitant radiosensitizing chemotherapy, remains the mainstay of therapy (56). In patients with stage IVB inoperable tumors or stage IVC disease, systemic therapy should be considered. Since 2017, the combination of dabrafenib + trametinib has been FDA approved for the treatment of BRAFV600E-mutated ATC and has revolutionized the management of these patients (176). Approval was based on results from the phase II ROAR basket trial, which showed an ORR of 56%, a median PFS of 6.7 months and a median OS of 14.5 months (28). However, real-life data showed far shorter OS with dabrafenib + trametinib alone (177, 178). One approach to potentially prolong duration of response in initially inoperable tumors has been to proceed with surgery after initial BRAF-directed therapy to make the tumor operable (57). In a retrospective study, this treatment strategy, known as the neoadjuvant approach, led to a 2-year OS of 80.3% in a population comprised of 63% stage IVC patients (179). In non-BRAF-mutated ATC, single agent kinase inhibitors have shorter responses, with a median PFS of 2.6 months with lenvatinib and 1.9 months with sorafenib (29, 30). In fact, under selective pressure, inherent genomic instability of ATC cells allows them to rapidly acquire escape mechanisms (163). Various potential mechanisms of resistance to therapy have been described in ATC, including activation of parallel pro-angiogenic signaling pathways, and acquisition of wildtype copy number amplification or secondary mutations in oncogenes such as RAS, NF1 and NF2 (180–185).

As previously detailed, ATC is suitable for immunotherapy with PD-1/PD-L1 inhibitors. Therefore, immune checkpoint inhibitors have been studied in this malignancy. However, once again, single-agent immunotherapy has shown limited efficacy. In a small retrospective study of 13 patients with ATC treated with anti-PD-1 monotherapy (pembrolizumab or nivolumab), ORR was 16% and median PFS 1.9 months (51). Similarly, in a prospective phase II trial, single-agent anti-PD-1 spartalizumab showed an ORR of 19% and a median PFS of 1.7 months (53). A phase I study combining durvalumab with tremelimumab and stereotactic body radiotherapy (SBRT) for metastatic ATC showed a median OS of only 14.5 weeks (49).

Although single-agent immunotherapy showed modest efficacy in ATC, combination of immune checkpoint inhibitors with kinase inhibitors had more promising results, owing to a synergistic effect between these two drug classes (186–188). In a murine model of BRAFV600E-mutant ATC, combination of a BRAF inhibitor with an anti-PD-L1 antibody led to significantly more tumor shrinkage compared to either agent alone (186). Similarly, in another murine model, anti-PD-1/PD-L1 immunotherapy was shown to enhance efficacy of lenvatinib in ATC (187). These observations were then supported by clinical data, showing prolonged responses to combinations of kinase inhibitors and immunotherapy in patients with ATC. In a retrospective study of 12 ATC patients who had progressed on prior kinase inhibitors, addition of pembrolizumab at time of progression led to further prolongation of survival, with a median OS of 6.9 months from the addition of immunotherapy (50). In BRAFV600E-mutated ATC, a retrospective study of 71 patients comparing dabrafenib/trametinib alone to dabrafenib/trametinib + pembrolizumab added either at baseline or at time of progression showed significant improvement in survival with the addition of anti-PD1 immunotherapy, with a median OS of 17 months with the triplet as opposed to 9 months with BRAF/MEK inhibitors alone (p=0.037) (189). PFS was also significantly improved when an anti-PD1 was added to the initial treatment regimen (Median PFS 11 vs 4 months; p=0.049). Similarly, a phase II clinical trial of the BRAF inhibitor vemurafenib + MEK inhibitor cobimetinib combined with the anti-PD-L1 atezolizumab in patients with BRAFV600E-mutated ATC showed remarkable response rates (ORR 72%) and a 24-month OS of 67% (median OS not reached) (48). Moreover, in non-BRAF mutated ATC, combination of lenvatinib with pembrolizumab in a prospective phase II trial of 27 patients showed an ORR of 52% and a median OS of 11 months, as opposed to a median OS of only 3.2 months with lenvatinib monotherapy (29, 52). The significant improvement in survival outcomes shown with the addition of anti-PD1 immunotherapy to kinase inhibitors in ATC led to the incorporation of pembrolizumab, as monotherapy or in combination with lenvatinib, to the 2024 National Comprehensive Cancer Network (NCCN) guidelines as a potential treatment option in selected patients with ATC (190).

Multiple clinical trials looking at various other immunotherapy combinations in ATC are currently underway, including the combination of cabozantinib + atezolizumab (NCT04400474) and pembrolizumab + docetaxel (NCT03360890), among others. In an ongoing phase II trial (NCT03246958) of the combination of ipilimumab + nivolumab, 3/10 enrolled ATC patients had profound partial responses, two of which lasted more than one year (13 and 26 months) (191). Pembrolizumab is also being studied in the adjuvant setting in patients with stage IVB disease after intensity-modulated radiation therapy (NCT05059470). Finally, like in advanced DTC, CAR-Ts are also under investigation in ATC. A trial assessing the safety and tolerability of autologous CAR-Ts targeting intracellular adhesion molecular-1 (ICAM-1) in advanced refractory PDTC and ATC is currently ongoing (NCT04420754).

## Conclusion

Despite the favorable prognosis for DTCs, treatment of most advanced/metastatic DTC and ATC patients remain a challenge, as none of the available targeted therapies are curative. In the last decade, the enhanced understanding of TC-specific molecular drivers has led to the development and FDA-approval of targeted therapies for advanced TCs. Although these treatment options have had promising outcomes, many advanced TC patients will eventually progress due to acquired resistance to therapy. Recently, ICIs have been explored as treatment modality in TCs to reinvigorate anti-tumor T cell function. While ICIs have shown enhancement in survival rates especially when used in conjunction with other treatment strategies, it is often accompanied by toxicities that can preclude patients from further therapy and ultimately lead to tumor progression and mortality. In addition to T cells, multiple immune components which have been implicated in thyroid tumorigenesis offer novel potential approaches for TC treatment such as NK cell-based immunotherapy, DC vaccines, and M2 TAM blockade, for example. Deeper knowledge of the immune milieu of thyroid cancer, strong predictive and prognostic biomarkers, and effective mechanism-rooted clinical trial strategies are needed to improve prognosis of aggressive thyroid cancers.

## Author contributions

NT: Conceptualization, Writing – original draft, Writing – review & editing. SH: Conceptualization, Writing – original draft, Writing – review & editing. RO: Conceptualization, Writing – original draft. RR: Writing – original draft. YH: Writing – review & editing. MP: Writing – original draft. SA: Writing – original draft. JC-M: Writing – review & editing. MZ: Writing – review & editing. SL: Writing – review & editing. SK: Conceptualization, Writing – review & editing. MC: Conceptualization, Writing – review & editing, Writing – original draft. RN: Conceptualization, Writing – review & editing.

## References

[1] Ibrahimpasic T Ghossein R Shah JP Ganly I. Poorly differentiated carcinoma of the thyroid gland: current status and future prospects. Thyroid. (2019) 29:311–21. doi: 10.1089/thy.2018.0509 PMC6437626 30747050

[2] Prete A Borges de Souza P Censi S Muzza M Nucci N Sponziello M. Update on fundamental mechanisms of thyroid cancer. Front Endocrinol. (2020) 11:102. doi: 10.3389/fendo.2020.00102 PMC7082927 32231639

[3] Kitahara CM Schneider AB. Epidemiology of thyroid cancer. Cancer Epidemiol Biomarkers Prev. (2022) 31:1284–97. doi: 10.1158/1055-9965.EPI-21-1440 PMC9473679 35775227

[4] Surveillance, Epidemiology, and End Results (SEER) Program. SEER*Stat database. Bethesda, MD: National Cancer Institute, DCCPS, Surveillance Research Program (2023).

[5] SEER Cancer Stat Facts. Thyroid cancer. Bethesda, MD: National Cancer Institute (2023).

[6] Maniakas A Dadu R Busaidy NL Wang JR Ferrarotto R Lu C. Evaluation of overall survival in patients with anaplastic thyroid carcinoma, 2000-2019. JAMA Oncol. (2020) 6:1397–404. doi: 10.1001/jamaoncol.2020.3362 PMC7411939 32761153

[7] Hamidi S Hofmann MC Iyer PC Cabanillas ME Hu MI Busaidy NL. Review article: new treatments for advanced differentiated thyroid cancers and potential mechanisms of drug resistance. Front Endocrinol (Lausanne). (2023) 14:1176731. doi: 10.3389/fendo.2023.1176731 37435488 PMC10331470

[8] U.S.F.D. Administration. 2011 Notifications. U.S Food Drug Administration. (2011).

[9] McFarland DC Misiukiewicz KJ. Sorafenib in radioactive iodine-refractory well-differentiated metastatic thyroid cancer. Onco Targets Ther. (2014) 7:1291–9. doi: 10.2147/OTT PMC4105272 25053887

[10] Nair A Lemery SJ Yang J Marathe A Zhao L Zhao H. FDA approval summary: lenvatinib for progressive, radio-iodine-refractory differentiated thyroid cancer. Clin Cancer Res. (2015) 21:5205–8. doi: 10.1158/1078-0432.CCR-15-1377 26324740

[11] U.S.F.D. Administration. FDA grants accelerated approval to dabrafenib in combination with trametinib for unresectable or metastatic solid tumors with BRAF V600E mutation. U.S Food Drug Administration. (2022).

[12] U.S.F.D. Administration. FDA approves cabozantinib for differentiated thyroid cancer. U.S Food Drug Administration. (2021).

[13] Duke ES Bradford D Marcovitz M Amatya AK Mishra-Kalyani PS Nguyen E. FDA approval summary: selpercatinib for the treatment of advanced RET fusion-positive solid tumors. Clin Cancer Res. (2023) 29:3573–8. doi: 10.1158/1078-0432.CCR-23-0459 PMC10524590 37265412

[14] Menicali E Guzzetti M Morelli S Moretti S Puxeddu E. Immune landscape of thyroid cancers: new insights. Front Endocrinol (Lausanne). (2020) 11:637826. doi: 10.3389/fendo.2020.637826 33986723 PMC8112200

[15] Jung KY Cho SW Kim YA Kim D Oh BC Park DJ. Cancers with higher density of tumor-associated macrophages were associated with poor survival rates. J Pathol Transl Med. (2015) 49:318–24. doi: 10.4132/jptm.2015.06.01 PMC4508569 26081823

[16] Chintakuntlawar AV Rumilla KM Smith CY Jenkins SM Foote RL Kasperbauer JL. Expression of PD-1 and PD-L1 in anaplastic thyroid cancer patients treated with multimodal therapy: results from a retrospective study. J Clin Endocrinol Metab. (2017) 102:1943–50. doi: 10.1210/jc.2016-3756 28324060

[17] Marabelle A Le DT Ascierto PA Di Giacomo AM De Jesus-Acosta A Delord JP. Efficacy of pembrolizumab in patients with noncolorectal high microsatellite instability/mismatch repair-deficient cancer: results from the phase II KEYNOTE-158 study. J Clin Oncol. (2020) 38:1–10. doi: 10.1200/JCO.19.02105 31682550 PMC8184060

[18] Khan SA Kurian P Mobley B Burks T Beg MS Ross JS. Relationship of anaplastic thyroid cancer high tumor mutation burden and MSI-H status with response to anti-PD1 monotherapy. J Clin Oncol. (2018) 36:e18114–4. doi: 10.1200/JCO.2018.36.15_suppl.e18114

[19] Wirth LJ Eigendorff E Capdevila J Paz-Ares LG Lin C-C Taylor MH. Phase I/II study of spartalizumab (PDR001), an anti-PD1 mAb, in patients with anaplastic thyroid cancer. J Clin Oncol. (2018) 36:6024–4. doi: 10.1200/JCO.2018.36.15_suppl.6024

[20] Brose MS Robinson BG Sherman SI Jarzab B Lin CC Vaisman F. Cabozantinib for previously treated radioiodine-refractory differentiated thyroid cancer: Updated results from the phase 3 COSMIC-311 trial. Cancer. (2022) 128:4203–12. doi: 10.1002/cncr.34493 PMC10092751 36259380

[21] Busaidy NL Konda B Wei L Wirth LJ Devine C Daniels GA. Dabrafenib versus dabrafenib + Trametinib in BRAF-mutated radioactive iodine refractory differentiated thyroid cancer: results of a randomized, phase 2, open-label multicenter trial. Thyroid. (2022) 32:1184–92. doi: 10.1089/thy.2022.0115 PMC9595631 35658604

[22] Demetri GD De Braud F Drilon A Siena S Patel MR Cho BC. Updated integrated analysis of the efficacy and safety of entrectinib in patients with NTRK fusion-positive solid tumors. Clin Cancer Res. (2022) 28:1302–12. doi: 10.1158/1078-0432.CCR-21-3597 PMC9365368 35144967

[23] Waguespack SG Drilon A Lin JJ Brose MS McDermott R Almubarak M. Efficacy and safety of larotrectinib in patients with TRK fusion-positive thyroid carcinoma. Eur J Endocrinol. (2022) 186:631–43. doi: 10.1530/EJE-21-1259 PMC9066591 35333737

[24] Schlumberger M Tahara M Wirth LJ Robinson B Brose MS Elisei R. Lenvatinib versus placebo in radioiodine-refractory thyroid cancer. N Engl J Med. (2015) 372:621–30. doi: 10.1056/NEJMoa1406470 25671254

[25] Subbiah V Hu MI Mansfield AS Taylor MH Schuler M Zhu VW. Pralsetinib in patients with advanced/metastatic rearranged during transfection (RET)-altered thyroid cancer: updated efficacy and safety data from the ARROW study. Thyroid. (2024) 34:26–40. doi: 10.1089/thy.2023.0363 38009200

[26] Wirth LJ Sherman E Robinson B Solomon B Kang H Lorch J. Efficacy of selpercatinib in RET-altered thyroid cancers. N Engl J Med. (2020) 383:825–35. 10.1056/NEJMoa2005651 PMC10777663 32846061

[27] Brose MS Nutting CM Jarzab B Elisei R Siena S Bastholt L. Sorafenib in radioactive iodine-refractory, locally advanced or metastatic differentiated thyroid cancer: a randomised, double-blind, phase 3 trial. Lancet. (2014) 384:319–28. doi: 10.1016/S0140-6736(14)60421-9 PMC4366116 24768112

[28] Subbiah V Kreitman RJ Wainberg ZA Cho JY Schellens JHM Soria JC. Dabrafenib plus trametinib in patients with BRAF V600E-mutant anaplastic thyroid cancer: updated analysis from the phase II ROAR basket study. Ann Oncol. (2022) 33:406–15. doi: 10.1016/j.annonc.2021.12.014 PMC9338780 35026411

[29] Wirth LJ Brose MS Sherman EJ Licitra L Schlumberger M Sherman SI. Open-label, single-arm, multicenter, phase II trial of lenvatinib for the treatment of patients with anaplastic thyroid cancer. J Clin Oncol. (2021) 39:2359–66. doi: 10.1200/JCO.20.03093 PMC8280094 33961488

[30] Savvides P Nagaiah G Lavertu P Fu P Wright JJ Chapman R. Phase II trial of sorafenib in patients with advanced anaplastic carcinoma of the thyroid. Thyroid. (2013) 23:600–4. doi: 10.1089/thy.2012.0103 PMC3643255 23113752

[31] van Velsen EFS Peeters RP Stegenga MT van Kemenade FJ van Ginhoven TM van Balkum M. Evaluating disease-specific survival prediction of risk stratification and TNM systems in differentiated thyroid cancer. J Clin Endocrinol Metab. (2022) 108:e267–74. doi: 10.1210/clinem/dgac721 PMC10188308 36508298

[32] Baloch ZW Asa SL Barletta JA Ghossein RA Juhlin CC Jung CK. Overview of the 2022 WHO classification of thyroid neoplasms. Endocrine Pathol. (2022) 33:27–63. doi: 10.1007/s12022-022-09707-3 35288841

[33] Xu B Ghossein R. Critical prognostic parameters in the anatomic pathology reporting of differentiated follicular cell-derived thyroid carcinoma. Cancers. (2019) 11:1100. doi: 10.3390/cancers11081100 31382401 PMC6721517

[34] Al-Hakami HA Alqahtani R Alahmadi A Almutairi D Algarni M Alandejani T. Thyroid nodule size and prediction of cancer: A study at tertiary care hospital in Saudi Arabia. Cureus. (2020) 12:e7478. doi: 10.7759/cureus.7478 32351856 PMC7188016

[35] Yang Z Heng Y Zhou J Tao L Cai W. Central and lateral neck involvement in papillary thyroid carcinoma patients with or without thyroid capsular invasion: A multi-center analysis. Front Endocrinol. (2023) 14. doi: 10.3389/fendo.2023.1138085 PMC10034063 36967774

[36] D'Avanzo A Treseler P Ituarte PH Wong M Streja L Greenspan FS. Follicular thyroid carcinoma: histology and prognosis. Cancer. (2004) 100:1123–9. doi: 10.1002/cncr.20081 15022277

[37] Seethala RR Baloch ZW Barletta JA Khanafshar E Mete O Sadow PM. Noninvasive follicular thyroid neoplasm with papillary-like nuclear features: a review for pathologists. Mod Pathol. (2018) 31:39–55. doi: 10.1038/modpathol.2017.130 29052599

[38] Rosario PW Mourão GF. Noninvasive follicular thyroid neoplasm with papillary-like nuclear features (NIFTP): a review for clinicians. Endocrine-Related Cancer. (2019) 26:R259–66. doi: 10.1530/ERC-19-0048 30913533

[39] Ashorobi D Lopez PP. Follicular thyroid cancer, statPearls. Treasure Island (FL: StatPearls Publishing LLC. (2024). 30969597

[40] Coca-Pelaz A Rodrigo JP Shah JP Sanabria A Al Ghuzlan A Silver CE. Hürthle cell carcinoma of the thyroid gland: systematic review and meta-analysis. Adv Ther. (2021) 38:5144–64. doi: 10.1007/s12325-021-01876-7 34423400

[41] Siegmund S Landa I Wong KS Barletta JA. Hürthle cell neoplasms. Diagn Histopathology. (2021) 27:231–9. doi: 10.1016/j.mpdhp.2021.03.001

[42] Wenter V Albert NL Unterrainer M Ahmaddy F Ilhan H Jellinek A. Clinical impact of follicular oncocytic (Hürthle cell) carcinoma in comparison with corresponding classical follicular thyroid carcinoma. Eur J Nucl Med Mol Imaging. (2021) 48:449–60. doi: 10.1007/s00259-020-04952-2 PMC7835150 32683470

[43] Zhou X Zheng Z Chen C Zhao B Cao H Li T. Clinical characteristics and prognostic factors of Hurthle cell carcinoma: a population based study. BMC Cancer. (2020) 20:407. doi: 10.1186/s12885-020-06915-0 32398118 PMC7216584

[44] Taylor M DG Thein K Larlot Y Khan S Goldschmidt J Lebellec L. Cabozantinib in combination with atezolizumab as first line thrapy in patients with radioaiodine-refractory differentiated thyroid: results from cohort of 18 of the phase 1B COSMIC-21 study. Thyroid. (2022) 32. doi: 10.1089/thy.2022.29137.abstracts

[45] Konda B SE Masarelli E Xia B Muzaffar J Morris J Ryder M. Cabozantinb in combination with nivolumab and ipilimumab in patients with radioactive iodine-refractory differentiated thyroid cancer whose cancer progressed after one prior VEGFR targeted therapy: interim results of a multicenter phase 2 NCI-ITOG trial (NCI#10240). Thyroid. (2022) 32.

[46] Mehnert JM Varga A Brose MS Aggarwal RR Lin CC Prawira A. Safety and antitumor activity of the anti-PD-1 antibody pembrolizumab in patients with advanced, PD-L1-positive papillary or follicular thyroid cancer. BMC Cancer. (2019) 19:196. doi: 10.1186/s12885-019-5380-3 30832606 PMC6399859

[47] Haugen B French J Worden FP Konda B Sherman EJ Dadu R. Lenvatinib plus pembrolizumab combination therapy in patients with radioiodine-refractory (RAIR), progressive differentiated thyroid cancer (DTC): Results of a multicenter phase II international thyroid oncology group trial. J Clin Oncol. (2020) 38:6512–2. doi: 10.1200/JCO.2020.38.15_suppl.6512

[48] Cabanillas M Busaidy N Zafereo M Gule-Monroe M Liu S Ferrarotto R. BRAF/MEK inhibitor plus immunotherapy for BRAFV600E-mutated anaplastic thyroid carcinoma. Thyroid. (2022) 32(S1). doi: 10.1089/thy.2022.29140.lb.abstracts

[49] Lee NY Riaz N Wu V Brinkman T Tsai CJ Zhi W. A pilot study of durvalumab (MEDI4736) with tremelimumab in combination with image-guided stereotactic body radiotherapy in the treatment of metastatic anaplastic thyroid cancer. Thyroid. (2022) 32:799–806. doi: 10.1089/thy.2022.0050 35521657 PMC9293682

[50] Iyer PC Dadu R Gule-Monroe M Busaidy NL Ferrarotto R Habra MA. Salvage pembrolizumab added to kinase inhibitor therapy for the treatment of anaplastic thyroid carcinoma. J Immunother Cancer. (2018) 6:68. doi: 10.1186/s40425-018-0378-y 29996921 PMC6042271

[51] Hatashima A Archambeau B Armbruster H Xu M Shah M Konda B. An evaluation of clinical efficacy of immune checkpoint inhibitors for patients with anaplastic thyroid carcinoma. Thyroid. (2022) 32:926–36. doi: 10.1089/thy.2022.0073 35583228

[52] Dierks C Ruf J Seufert J Kreissl M Klein C Spitzweg C. 1646MO - Phase II ATLEP trial: Final results for lenvatinib/pembrolizumab in metastasized anaplastic and poorly differentiated thyroid carcinoma. Ann Oncol. (2022) 33. doi: 10.1016/j.annonc.2022.07.1726

[53] Capdevila J Wirth LJ Ernst T Ponce Aix S Lin CC Ramlau R. PD-1 blockade in anaplastic thyroid carcinoma. J Clin Oncol. (2020) 38:2620–7. doi: 10.1200/JCO.19.02727 PMC7476256 32364844

[54] Xu B David J Dogan S Landa I Katabi N Saliba M. Primary high-grade non-anaplastic thyroid carcinoma: a retrospective study of 364 cases. Histopathology. (2022) 80:322–37. doi: 10.1111/his.14550 PMC9425734 34449926

[55] Pozdeyev N Gay LM Sokol ES Hartmaier R Deaver KE Davis S. Genetic analysis of 779 advanced differentiated and anaplastic thyroid cancers. Clin Cancer Res. (2018) 24:3059–68. doi: 10.1158/1078-0432.CCR-18-0373 PMC6030480 29615459

[56] Bible KC Kebebew E Brierley J Brito JP Cabanillas ME Clark TJ Jr.. 2021 American thyroid association guidelines for management of patients with anaplastic thyroid cancer. Thyroid. (2021) 31:337–86. doi: 10.1089/thy.2020.0944 PMC8349723 33728999

[57] Cabanillas ME Ferrarotto R Garden AS Ahmed S Busaidy NL Dadu R. and immune-directed therapy for anaplastic thyroid carcinoma. Thyroid. (2018) 28:945–51. doi: 10.1089/thy.2018.0060 PMC6425982 29742974

[58] Filetti S Durante C Hartl D Leboulleux S Locati LD Newbold K. Thyroid cancer: ESMO Clinical Practice Guidelines for diagnosis, treatment and follow-up†. Ann Oncol. (2019) 30:1856–83. doi: 10.1093/annonc/mdz400 31549998

[59] Shao C Li G Huang L Pruitt S Castellanos E Frampton G. Prevalence of high tumor mutational burden and association with survival in patients with less common solid tumors. JAMA Network Open. (2020) 3:e2025109–e2025109. doi: 10.1001/jamanetworkopen.2020.25109 33119110 PMC7596577

[60] Hu J Yuan IJ Mirshahidi S Simental A Lee SC Yuan X. Thyroid carcinoma: phenotypic features, underlying biology and potential relevance for targeting therapy. Int J Mol Sci. (2021) 22:1950. doi: 10.3390/ijms22041950 33669363 PMC7920269

[61] Schubert L Mariko ML Clerc J Huillard O Groussin L. MAPK pathway inhibitors in thyroid cancer: preclinical and clinical data. Cancers (Basel). (2023) 15:710. doi: 10.3390/cancers15030710 36765665 PMC9913385

[62] Bikas A Ahmadi S Pappa T Marqusee E Wong K Nehs MA. Additional oncogenic alterations in RAS-driven differentiated thyroid cancers associate with worse clinicopathologic outcomes. Clin Cancer Res. (2023) 29:2678–85. doi: 10.1158/1078-0432.CCR-23-0278 PMC10524472 37260297

[63] Cheng L Newbold K. Genomic landscape of anaplastic thyroid cancer and implications on therapy. Curr Opin Endocrine Metab Res. (2023) 30:100458. doi: 10.1016/j.coemr.2023.100458

[64] Agrawal N Akbani R Arman AB Ally A Arachchi H Asa SL. Integrated genomic characterization of papillary thyroid carcinoma. Cell. (2014) 159:676–90. doi: 10.1016/j.cell.2014.09.050 PMC4243044 25417114

[65] Chakravarty D Santos E Ryder M Knauf JA Liao X-H West BL. Small-molecule MAPK inhibitors restore radioiodine incorporation in mouse thyroid cancers with conditional BRAF activation. J Clin Invest. (2011) 121:4700–11. doi: 10.1172/JCI46382 PMC3225989 22105174

[66] Oh JM Ahn BC. Molecular mechanisms of radioactive iodine refractoriness in differentiated thyroid cancer: Impaired sodium iodide symporter (NIS) expression owing to altered signaling pathway activity and intracellular localization of NIS. Theranostics. (2021) 11:6251–77. doi: 10.7150/thno.57689 PMC8120202 33995657

[67] Boucai L Saqcena M Kuo F Grewal RK Socci N Knauf JA. Genomic and transcriptomic characteristics of metastatic thyroid cancers with exceptional responses to radioactive iodine therapy. Clin Cancer Res. (2023) 29:1620–30. doi: 10.1158/1078-0432.CCR-22-2882 PMC10106408 36780190

[68] Landa I Ibrahimpasic T Boucai L Sinha R Knauf JA Shah RH. Genomic and transcriptomic hallmarks of poorly differentiated and anaplastic thyroid cancers. J Clin Invest. (2016) 126:1052–66. doi: 10.1172/JCI85271 PMC4767360 26878173

[69] Yoo SK Lee S Kim SJ Jee HG Kim BA Cho H. Comprehensive analysis of the transcriptional and mutational landscape of follicular and papillary thyroid cancers. PloS Genet. (2016) 12:e1006239. doi: 10.1371/journal.pgen.1006239 27494611 PMC4975456

[70] Cao J Zhu X Sun Y Li X Yun C Zhang W. The genetic duet of BRAF V600E and TERT promoter mutations predicts the poor curative effect of radioiodine therapy in papillary thyroid cancer. Eur J Nucl Med Mol Imaging. (2022) 49:3470–81. doi: 10.1007/s00259-022-05820-x 35501518

[71] Yu P Qu N Zhu R Hu J Han P Wu J. TERT accelerates BRAF mutant-induced thyroid cancer dedifferentiation and progression by regulating ribosome biogenesis. Sci Adv. (2023) 9:eadg7125. doi: 10.1126/sciadv.adg7125 37647391 PMC10468137

[72] Cabanillas ME Ryder M Jimenez C. Targeted therapy for advanced thyroid cancer: kinase inhibitors and beyond. Endocr Rev. (2019) 40:1573–604. doi: 10.1210/er.2019-00007 PMC7341904 31322645

[73] Ahmadi S Landa I. The prognostic power of gene mutations in thyroid cancer. Endocrine Connections. (2024) 13:e230297. doi: 10.1530/EC-23-0297 38078934 PMC10831542

[74] Boos LA Dettmer M Schmitt A Rudolph T Steinert H Moch H. Diagnostic and prognostic implications of the PAX8-PPARγ translocation in thyroid carcinomas-a TMA-based study of 226 cases. Histopathology. (2013) 63:234–41. doi: 10.1111/his.12150 23738683

[75] Yoo S-K Song YS Lee EK Hwang J Kim HH Jung G. Integrative analysis of genomic and transcriptomic characteristics associated with progression of aggressive thyroid cancer. Nat Commun. (2019) 10:2764. doi: 10.1038/s41467-019-10680-5 31235699 PMC6591357

[76] Nicolson NG Murtha TD Dong W Paulsson JO Choi J Barbieri AL. Comprehensive genetic analysis of follicular thyroid carcinoma predicts prognosis independent of histology. J Clin Endocrinol Metab. (2018) 103:2640–50. doi: 10.1210/jc.2018-00277 29726952

[77] Ganly I Makarov V Deraje S Dong Y Reznik E Seshan V. Integrated genomic analysis of hürthle cell cancer reveals oncogenic drivers, recurrent mitochondrial mutations, and unique chromosomal landscapes. Cancer Cell. (2018) 34:256–270.e255. doi: 10.1016/j.ccell.2018.07.002 30107176 PMC6247912

[78] Wang JR Montierth M Xu L Goswami M Zhao X Cote G. Impact of somatic mutations on survival outcomes in patients with anaplastic thyroid carcinoma. JCO Precis Oncol. (2022) 6:e2100504. doi: 10.1200/PO.21.00504 35977347 PMC10530586

[79] Capdevila J Mayor R Mancuso F Iglesias C Caratù G Matos I. Early evolutionary divergence between papillary and anaplastic thyroid cancers. Ann Oncol. (2018) 29:1454–60. doi: 10.1093/annonc/mdy123 29648575

[80] Shin E Koo JS. Cell component and function of tumor microenvironment in thyroid cancer. Int J Mol Sci. (2022) 23:12578. doi: 10.3390/ijms232012578 36293435 PMC9604510

[81] Giannini R Moretti S Ugolini C Macerola E Menicali E Nucci N. Immune profiling of thyroid carcinomas suggests the existence of two major phenotypes: an ATC-like and a PDTC-like. J Clin Endocrinol Metab. (2019) 104:3557–75. doi: 10.1210/jc.2018-01167 30882858

[82] Zhu L Li XJ Kalimuthu S Gangadaran P Lee HW Oh JM. Natural killer cell (NK-92MI)-based therapy for pulmonary metastasis of anaplastic thyroid cancer in a nude mouse model. Front Immunol. (2017) 8. doi: 10.3389/fimmu.2017.00816 PMC5519537 28785259

[83] Lu L Wang JR Henderson YC Bai S Yang J Hu M. Anaplastic transformation in thyroid cancer revealed by single-cell transcriptomics. J Clin Invest. (2023) 133:e169653. doi: 10.1172/JCI169653 37053016 PMC10231997

[84] Fozzatti L Cheng SY. Tumor cells and cancer-associated fibroblasts: A synergistic crosstalk to promote thyroid cancer. Endocrinol Metab (Seoul). (2020) 35:673–80. doi: 10.3803/EnM.2020.401 PMC7803596 33161690

[85] Fozzatti L Alamino VA Park S Giusiano L Volpini X Zhao L. Interplay of fibroblasts with anaplastic tumor cells promotes follicular thyroid cancer progression. Sci Rep. (2019) 9:8028. doi: 10.1038/s41598-019-44361-6 31142771 PMC6541589

[86] Minna E Brich S Todoerti K Pilotti S Collini P Bonaldi E. Cancer associated fibroblasts and senescent thyroid cells in the invasive front of thyroid carcinoma. Cancers (Basel). (2020) 12:112. doi: 10.3390/cancers12010112 31906302 PMC7016563

[87] Yang Z Wei X Pan Y Xu J Si Y Min Z. A new risk factor indicator for papillary thyroid cancer based on immune infiltration. Cell Death Dis. (2021) 12:51. doi: 10.1038/s41419-020-03294-z 33414407 PMC7791058

[88] Wen S Qu N Ma B Wang X Luo Y Xu W. Cancer-associated fibroblasts positively correlate with dedifferentiation and aggressiveness of thyroid cancer. Onco Targets Ther. (2021) 14:1205–17. doi: 10.2147/OTT.S294725 PMC7910116 33654411

[89] Ryder M Ghossein RA Ricarte-Filho JC Knauf JA Fagin JA. Increased density of tumor-associated macrophages is associated with decreased survival in advanced thyroid cancer. Endocr Relat Cancer. (2008) 15:1069–74. doi: 10.1677/ERC-08-0036 PMC2648614 18719091

[90] Caillou B Talbot M Weyemi U Pioche-Durieu C Al Ghuzlan A Bidart JM. Tumor-associated macrophages (TAMs) form an interconnected cellular supportive network in anaplastic thyroid carcinoma. PloS One. (2011) 6:e22567. doi: 10.1371/journal.pone.0022567 21811634 PMC3141071

[91] Kim S Cho SW Min HS Kim KM Yeom GJ Kim EY. The expression of tumor-associated macrophages in papillary thyroid carcinoma. Endocrinol Metab (Seoul). (2013) 28:192–8. doi: 10.3803/EnM.2013.28.3.192 PMC3811699 24396678

[92] Qing W Fang WY Ye L Shen LY Zhang XF Fei XC. Density of tumor-associated macrophages correlates with lymph node metastasis in papillary thyroid carcinoma. Thyroid. (2012) 22:905–10. doi: 10.1089/thy.2011.0452 PMC3429273 22870901

[93] Kim DI Kim E Kim YA Cho SW Lim JA Park YJ. Macrophage densities correlated with CXC chemokine receptor 4 expression and related with poor survival in anaplastic thyroid cancer. Endocrinol Metab (Seoul). (2016) 31:469–75. doi: 10.3803/EnM.2016.31.3.469 PMC5053061 27491720

[94] Palacios LM Peyret V Viano ME Geysels RC Chocobar YA Volpini X. TIM3 expression in anaplastic-thyroid-cancer-infiltrating macrophages: an emerging immunotherapeutic target. Biol (Basel) 11. (2022) 11(11):1609. doi: 10.3390/biology11111609 PMC9687546 36358310

[95] Luo Y Yang YC Ma B Xu WB Liao T Wang Y. Integrated analysis of novel macrophage related signature in anaplastic thyroid cancer. Endocrine. (2022) 78:517–30. doi: 10.1007/s12020-022-03179-5 36070052

[96] Cho JW Kim WW Lee YM Jeon MJ Kim WG Song DE. Impact of tumor-associated macrophages and BRAF(V600E) mutation on clinical outcomes in patients with various thyroid cancers. Head Neck. (2019) 41:686–91. doi: 10.1002/hed.25469 30659691

[97] Suzuki S Shibata M Gonda K Kanke Y Ashizawa M Ujiie D. Immunosuppression involving increased myeloid-derived suppressor cell levels, systemic inflammation and hypoalbuminemia are present in patients with anaplastic thyroid cancer. Mol Clin Oncol. (2013) 1:959–64. doi: 10.3892/mco.2013.170 PMC3915656 24649277

[98] Condamine T Ramachandran I Youn JI Gabrilovich DI. Regulation of tumor metastasis by myeloid-derived suppressor cells. Annu Rev Med. (2015) 66:97–110. doi: 10.1146/annurev-med-051013-052304 25341012 PMC4324727

[99] Wen L Gong P Liang C Shou D Liu B Chen Y. Interplay between myeloid-derived suppressor cells (MDSCs) and Th17 cells: foe or friend? Oncotarget. (2016) 7:35490–6. doi: 10.18632/oncotarget.v7i23 PMC5085246 27007054

[100] Xu B Zhang L Setoodeh R Mohanty AS Landa I Balzer B. Prolonged survival of anaplastic thyroid carcinoma is associated with resectability, low tumor-infiltrating neutrophils/myeloid-derived suppressor cells, and low peripheral neutrophil-to-lymphocyte ratio. Endocrine. (2022) 76:612–9. doi: 10.1007/s12020-022-03008-9 PMC10173871 35149932

[101] Tran Janco JM Lamichhane P Karyampudi L Knutson KL. Tumor-infiltrating dendritic cells in cancer pathogenesis. J Immunol. (2015) 194:2985–91. doi: 10.4049/jimmunol.1403134 PMC4369768 25795789

[102] Harimoto H Shimizu M Nakagawa Y Nakatsuka K Wakabayashi A Sakamoto C. Inactivation of tumor-specific CD8⁺ CTLs by tumor-infiltrating tolerogenic dendritic cells. Immunol Cell Biol. (2013) 91:545–55. doi: 10.1038/icb.2013.38 PMC3806489 24018532

[103] Hilly O Koren R Raz R Rath-Wolfson L Mizrachi A Hamzany Y. The role of s100-positive dendritic cells in the prognosis of papillary thyroid carcinoma. Am J Clin Pathol. (2013) 139:87–92. doi: 10.1309/AJCPAKYDO56NKMYZ 23270903

[104] Ugolini C Basolo F Proietti A Vitti P Elisei R Miccoli P. Lymphocyte and immature dendritic cell infiltrates in differentiated, poorly differentiated, and undifferentiated thyroid carcinoma. Thyroid. (2007) 17:389–93. doi: 10.1089/thy.2006.0306 17542668

[105] Kuwabara K Nishishita T Morishita M Oyaizu N Yamashita S Kanematsu T. Results of a phase I clinical study using dendritic cell vaccinations for thyroid cancer. Thyroid. (2007) 17:53–8. doi: 10.1089/thy.2006.0178 17274750

[106] Koga Y Matsuzaki A Suminoe A Hattori H Hara T. Neutrophil-derived TNF-related apoptosis-inducing ligand (TRAIL): a novel mechanism of antitumor effect by neutrophils. Cancer Res. (2004) 64:1037–43. doi: 10.1158/0008-5472.CAN-03-1808 14871835

[107] Mahiddine K Blaisdell A Ma S Créquer-Grandhomme A Lowell CA Erlebacher A. Relief of tumor hypoxia unleashes the tumoricidal potential of neutrophils. J Clin Invest. (2020) 130:389–403. doi: 10.1172/JCI130952 31600172 PMC6934192

[108] Finisguerra V Di Conza G Di Matteo M Serneels J Costa S Thompson AA. MET is required for the recruitment of anti-tumoural neutrophils. Nature. (2015) 522:349–53. doi: 10.1038/nature14407 PMC4594765 25985180

[109] Knaapen AM Seiler F Schilderman PA Nehls P Bruch J Schins RP. Neutrophils cause oxidative DNA damage in alveolar epithelial cells. Free Radic Biol Med. (1999) 27:234–40. doi: 10.1016/S0891-5849(98)00285-8 10443941

[110] Canli Ö. Nicolas AM Gupta J Finkelmeier F Goncharova O Pesic M. Myeloid cell-derived reactive oxygen species induce epithelial mutagenesis. Cancer Cell. (2017) 32:869–883.e865. doi: 10.1016/j.ccell.2017.11.004 29232557

[111] Coussens LM Werb Z. Inflammation and cancer. Nature. (2002) 420:860–7. doi: 10.1038/nature01322 PMC2803035 12490959

[112] Cho MJ Park KS Cho MJ Yoo YB Yang JH. A comparative analysis of endoscopic thyroidectomy versus conventional thyroidectomy in clinically lymph node negative thyroid cancer. Ann Surg Treat Res. (2015) 88:69–76. doi: 10.4174/astr.2015.88.2.69 25692117 PMC4325647

[113] Cho JS Park MH Ryu YJ Yoon JH. The neutrophil to lymphocyte ratio can discriminate anaplastic thyroid cancer against poorly or well differentiated cancer. Ann Surg Treat Res. (2015) 88:187–92. doi: 10.4174/astr.2015.88.4.187 PMC4384286 25844352

[114] Cristinziano L Modestino L Loffredo S Varricchi G Braile M Ferrara AL. Anaplastic thyroid cancer cells induce the release of mitochondrial extracellular DNA traps by viable neutrophils. J Immunol. (2020) 204:1362–72. doi: 10.4049/jimmunol.1900543 31959732

[115] Yin M Di G Bian M. Dysfunction of natural killer cells mediated by PD-1 and Tim-3 pathway in anaplastic thyroid cancer. Int Immunopharmacol. (2018) 64:333–9. doi: 10.1016/j.intimp.2018.09.016 30243069

[116] Poli A Michel T Thérésine M Andrès E Hentges F Zimmer J. CD56bright natural killer (NK) cells: an important NK cell subset. Immunology. (2009) 126:458–65. doi: 10.1111/j.1365-2567.2008.03027.x PMC2673358 19278419

[117] Gogali F Paterakis G Rassidakis GZ Liakou CI Liapi C. CD3(-)CD16(-)CD56(bright) immunoregulatory NK cells are increased in the tumor microenvironment and inversely correlate with advanced stages in patients with papillary thyroid cancer. Thyroid. (2013) 23:1561–8. doi: 10.1089/thy.2012.0560 23721357

[118] Wennerberg E Pfefferle A Ekblad L Yoshimoto Y Kremer V Kaminskyy VO. Human anaplastic thyroid carcinoma cells are sensitive to NK cell-mediated lysis via ULBP2/5/6 and chemoattract NK cells. Clin Cancer Res. (2014) 20:5733–44. doi: 10.1158/1078-0432.CCR-14-0291 25212604

[119] Zhang L Conejo-Garcia JR Katsaros D Gimotty PA Massobrio M Regnani G. Intratumoral T cells, recurrence, and survival in epithelial ovarian cancer. N Engl J Med. (2003) 348:203–13. doi: 10.1056/NEJMoa020177 12529460

[120] Teng F Meng X Kong L Mu D Zhu H Liu S. Tumor-infiltrating lymphocytes, forkhead box P3, programmed death ligand-1, and cytotoxic T lymphocyte–associated antigen-4 expressions before and after neoadjuvant chemoradiation in rectal cancer. Trans Res. (2015) 166:721–732.e721. doi: 10.1016/j.trsl.2015.06.019 26209749

[121] Clemente CG Mihm MC Jr. Bufalino R Zurrida S Collini P Cascinelli N. Prognostic value of tumor infiltrating lymphocytes in the vertical growth phase of primary cutaneous melanoma. Cancer. (1996) 77:1303–10. doi: 10.1002/(SICI)1097-0142(19960401)77:7<1303::AID-CNCR12>3.0.CO;2-5 8608507

[122] Sato E Olson SH Ahn J Bundy B Nishikawa H Qian F. Intraepithelial CD8+ tumor-infiltrating lymphocytes and a high CD8+/regulatory T cell ratio are associated with favorable prognosis in ovarian cancer. Proc Natl Acad Sci U.S.A. (2005) 102:18538–43. doi: 10.1073/pnas.0509182102 PMC1311741 16344461

[123] Galon J Costes A Sanchez-Cabo F Kirilovsky A Mlecnik B Lagorce-Pagès C. Type, density, and location of immune cells within human colorectal tumors predict clinical outcome. Science. (2006) 313:1960–4. doi: 10.1126/science.1129139 17008531

[124] Cunha LL Morari EC Guihen AC Razolli D Gerhard R Nonogaki S. Infiltration of a mixture of immune cells may be related to good prognosis in patients with differentiated thyroid carcinoma. Clin Endocrinol (Oxf). (2012) 77:918–25. doi: 10.1111/j.1365-2265.2012.04482.x 22738343

[125] Cunha LL Marcello MA Nonogaki S Morari EC Soares FA Vassallo J. CD8+ tumour-infiltrating lymphocytes and COX2 expression may predict relapse in differentiated thyroid cancer. Clin Endocrinol (Oxf). (2015) 83:246–53. doi: 10.1111/cen.12586 25130519

[126] Severson JJ Serracino HS Mateescu V Raeburn CD McIntyre RC Jr. Sams SB. PD-1+Tim-3+ CD8+ T lymphocytes display varied degrees of functional exhaustion in patients with regionally metastatic differentiated thyroid cancer. Cancer Immunol Res. (2015) 3:620–30. doi: 10.1158/2326-6066.CIR-14-0201 PMC4457654 25701326

[127] Zhou Y Guo L Sun H Xu J Ba T. CXCR5(+) CD8 T cells displayed higher activation potential despite high PD-1 expression, in tumor-involved lymph nodes from patients with thyroid cancer. Int Immunopharmacol. (2018) 62:114–9. doi: 10.1016/j.intimp.2018.07.002 30005226

[128] Haugen BR Alexander EK Bible KC Doherty GM Mandel SJ Nikiforov YE. 2015 American thyroid association management guidelines for adult patients with thyroid nodules and differentiated thyroid cancer: the American thyroid association guidelines task force on thyroid nodules and differentiated thyroid cancer. Thyroid. (2016) 26:1–133. doi: 10.1089/thy.2015.0020 26462967 PMC4739132

[129] Matsuzu K Sugino K Masudo K Nagahama M Kitagawa W Shibuya H. Thyroid lobectomy for papillary thyroid cancer: long-term follow-up study of 1,088 cases. World J Surg. (2014) 38:68–79. doi: 10.1007/s00268-013-2224-1 24081532

[130] Nixon IJ Ganly I Patel SG Palmer FL Whitcher MM Tuttle RM. Thyroid lobectomy for treatment of well differentiated intrathyroid Malignancy. Surgery. (2012) 151:571–9. doi: 10.1016/j.surg.2011.08.016 22001636

[131] Fugazzola L Elisei R Fuhrer D Jarzab B Leboulleux S Newbold K. 2019 European thyroid association guidelines for the treatment and follow-Up of advanced radioiodine-Refractory thyroid cancer. Eur Thyroid J. (2019) 8:227–45. doi: 10.1159/000502229 PMC6873012 31768334

[132] Haugen BR. 2015 American Thyroid Association Management Guidelines for Adult Patients with Thyroid Nodules and Differentiated Thyroid Cancer: What is new and what has changed? Cancer. (2017) 123:372–81. doi: 10.1002/cncr.30360 27741354

[133] Aashiq M Silverman DA Na'ara S Takahashi H Amit M. Radioiodine-refractory thyroid cancer: molecular basis of redifferentiation therapies, management, and novel therapies. Cancers (Basel). (2019) 11(9):1382. doi: 10.3390/cancers11091382 31533238 PMC6770909

[134] Filetti S Durante C Hartl DM Leboulleux S Locati LD Newbold K. ESMO Clinical Practice Guideline update on the use of systemic therapy in advanced thyroid cancer. Ann Oncol. (2022) 33:674–84. E.G.C.E.a. clinicalguidelines@esmo.org. doi: 10.1016/j.annonc.2022.04.009 35491008

[135] Shojaei F Lee JH Simmons BH Wong A Esparza CO Plumlee PA. HGF/c-Met acts as an alternative angiogenic pathway in sunitinib-resistant tumors. Cancer Res. (2010) 70:10090–100. doi: 10.1158/0008-5472.CAN-10-0489 20952508

[136] Zhou L Liu XD Sun M Zhang X German P Bai S. and AXL overcomes resistance to sunitinib therapy in renal cell carcinoma. Oncogene. (2016) 35:2687–97. doi: 10.1038/onc.2015.343 PMC4791213 26364599

[137] Fagin JA Wells SA Jr. Biologic and clinical perspectives on thyroid cancer. N Engl J Med. (2016) 375:2307. doi: 10.1056/NEJMc1613118 27959677

[138] Aydemirli MD van Eendenburg JDH van Wezel T Oosting J Corver WE Kapiteijn E. Targeting EML4-ALK gene fusion variant 3 in thyroid cancer. Endocrine-Related Cancer. (2021) 28:377–89. doi: 10.1530/ERC-20-0436 PMC8183637 33878728

[139] Zhu L Ma S Xia B. Remarkable response to alectinib for metastatic papillary thyroid cancer with STRN-ALK fusion: A case report. Front Oncol. (2022) 12:1009076. doi: 10.3389/fonc.2022.1009076 36439495 PMC9682147

[140] Lamartina L Anizan N Dupuy C Leboulleux S Schlumberger M. Redifferentiation-facilitated radioiodine therapy in thyroid cancer. Endocr Relat Cancer. (2021) 28:T179–91. doi: 10.1530/ERC-21-0024 33690158

[141] Ho AL Grewal RK Leboeuf R Sherman EJ Pfister DG Deandreis D. Selumetinib-enhanced radioiodine uptake in advanced thyroid cancer. N Engl J Med. (2013) 368:623–32. doi: 10.1056/NEJMoa1209288 PMC3615415 23406027

[142] Jaber T Waguespack SG Cabanillas ME Elbanan M Vu T Dadu R. Targeted therapy in advanced thyroid cancer to resensitize tumors to radioactive iodine. J Clin Endocrinol Metab. (2018) 103:3698–705. doi: 10.1210/jc.2018-00612 PMC6179172 30032208

[143] Iravani A Solomon B Pattison DA Jackson P Ravi Kumar A Kong G. Mitogen-activated protein kinase pathway inhibition for redifferentiation of radioiodine refractory differentiated thyroid cancer: an evolving protocol. Thyroid. (2019) 29:1634–45. doi: 10.1089/thy.2019.0143 31637953

[144] Leboulleux S Benisvy D Taieb D Attard M Bournaud C Terroir-Cassou-Mounat M. MERAIODE: A phase II redifferentiation trial with trametinib and (131)I in metastatic radioactive iodine refractory RAS mutated differentiated thyroid cancer. Thyroid. (2023) 33:1124–9. doi: 10.1089/thy.2023.0240 37350119

[145] Burman B Tuttle RM Grewal RK Sherman EJ Baxi SS Boucai L. Phase 2 of trametinib plus radioiodine in RAS-mutant and wild-type, radioiodine-refractory thyroid cancer (ETCTN9446). J Clin Oncol. (2022) 40:6089–9. doi: 10.1200/JCO.2022.40.16_suppl.6089

[146] Leboulleux S Do Cao C Zerdoud S Attard M Bournaud C Lacroix L. A Phase II Redifferentiation Trial with Dabrafenib-Trametinib and 131I in Metastatic Radioactive Iodine Refractory BRAF p.V600E Mutated Differentiated thyroid Cancer. Clin Cancer Res. (2023) 29(13):2401–9. doi: 10.1158/1078-0432.CCR-23-0046 37074727

[147] Dunn LA Sherman EJ Baxi SS Tchekmedyian V Grewal RK Larson SM. Vemurafenib redifferentiation of BRAF mutant, RAI-refractory thyroid cancers. J Clin Endocrinol Metab. (2019) 104:1417–28. doi: 10.1210/jc.2018-01478 PMC6435099 30256977

[148] Rothenberg SM Daniels GH Wirth LJ. Redifferentiation of iodine-refractory BRAF V600E-mutant metastatic papillary thyroid cancer with dabrafenib-response. Clin Cancer Res. (2015) 21:5640–1. doi: 10.1158/1078-0432.CCR-15-2298 26672087

[149] Weber M Kersting D Riemann B Brandenburg T Fuhrer-Sakel D Grunwald F. Enhancing radioiodine incorporation into radioiodine-refractory thyroid cancer with MAPK inhibition (ERRITI): A single-center prospective two-arm study. Clin Cancer Res. (2022) 28:4194–202. doi: 10.1158/1078-0432.CCR-22-0437 PMC9527501 35594174

[150] Groussin L Clerc J Huillard O. Larotrectinib-enhanced radioactive iodine uptake in advanced thyroid cancer. N Engl J Med. (2020) 383:1686–7. doi: 10.1056/NEJMc2023094 33085869

[151] Chan HP Chen IF Tsai FR Kao CH Shen DH. Reversing "Flip-flop" Phenomenon of 131 I and glucose avidity in RET-fusion positive radioiodine-refractory thyroid cancer lesions after treatment of pralsetinib. Clin Nucl Med. (2023) 48:e147–8. doi: 10.1097/RLU.0000000000004475 36327463

[152] Lee YA Lee H Im SW Song YS Oh DY Kang HJ. NTRK and RET fusion-directed therapy in pediatric thyroid cancer yields a tumor response and radioiodine uptake. J Clin Invest. (2021) 131:e144847. doi: 10.1172/JCI144847 34237031 PMC8439610

[153] Waguespack SG Tewari SO Busaidy NL Zafereo ME. Larotrectinib before initial radioactive iodine therapy in pediatric TRK fusion-positive papillary thyroid carcinoma: time to reconsider the treatment paradigm for distantly metastatic disease? JCO Precis Oncol. (2022) 6:e2100467. doi: 10.1200/PO.21.00467 35420905 PMC9029926

[154] Cabanillas ME Busaidy NL Sherman SI. Redifferentiation therapy-returning to our roots in a post-kinase inhibitor world. Clin Cancer Res. (2022) 28:4164–6. doi: 10.1158/1078-0432.CCR-22-1710 35895318

[155] Toro-Tobon D Morris JC Hilger C Peskey C Durski JM Ryder M. Clinical outcomes of radioactive iodine redifferentiation therapy in previously iodine refractory differentiated thyroid cancers. Thyroid. (2024) 34:70–81. doi: 10.1089/thy.2023.0456 37917101

[156] Hatzivassiliou G Song K Yen I Brandhuber BJ Anderson DJ Alvarado R. RAF inhibitors prime wild-type RAF to activate the MAPK pathway and enhance growth. Nature. (2010) 464:431–5. doi: 10.1038/nature08833 20130576

[157] Jin T Lavoie H Sahmi M David M Hilt C Hammell A. RAF inhibitors promote RAS-RAF interaction by allosterically disrupting RAF autoinhibition. Nat Commun. (2017) 8:1211. doi: 10.1038/s41467-017-01274-0 29084939 PMC5662619

[158] Rasco DW Medina T Corrie P Pavlick AC Middleton MR Lorigan P. Phase 1 study of the pan-RAF inhibitor tovorafenib in patients with advanced solid tumors followed by dose expansion in patients with metastatic melanoma. Cancer Chemother Pharmacol. (2023) 92:15–28. doi: 10.1007/s00280-023-04544-5 37219686 PMC10261210

[159] Yap JL Worlikar S MacKerell AD Jr. Shapiro P Fletcher S. Small-molecule inhibitors of the ERK signaling pathway: Towards novel anticancer therapeutics. ChemMedChem. (2011) 6:38–48. doi: 10.1002/cmdc.201000354 21110380 PMC3477473

[160] Sullivan RJ Infante JR Janku F Wong DJL Sosman JA Keedy V. First-in-class ERK1/2 inhibitor ulixertinib (BVD-523) in patients with MAPK mutant advanced solid tumors: results of a phase I dose-escalation and expansion study. Cancer Discovery. (2018) 8:184–95. doi: 10.1158/2159-8290.CD-17-1119 29247021

[161] Taza F Durm GA Opyrchal M Jalal SI Radovich M Schneider BP. A phase 2 basket trial of an ERK1/2 inhibitor (LY3214996) in combination with abemaciclib for patients whose tumors harbor pathogenic alterations in BRAF, RAF1, MAP2K1/2 ERK1/2, and NF1. J Clin Oncol. (2023) 41:e15088–8. doi: 10.1200/JCO.2023.41.16_suppl.e15088

[162] Śmiech M Leszczyński P Kono H Wardell C Taniguchi H. Emerging BRAF mutations in cancer progression and their possible effects on transcriptional networks. Genes (Basel). (2020) 11:1342. doi: 10.3390/genes11111342 33198372 PMC7697059

[163] Hofmann MC Kunnimalaiyaan M Wang JR Busaidy NL Sherman SI Lai SY. Molecular mechanisms of resistance to kinase inhibitors and redifferentiation in thyroid cancers. Endocr Relat Cancer. (2022) 29:R173–90. doi: 10.1530/ERC-22-0129 PMC9534048 35975971

[164] Zhang C Spevak W Zhang Y Burton EA Ma Y Habets G. RAF inhibitors that evade paradoxical MAPK pathway activation. Nature. (2015) 526:583–6. doi: 10.1038/nature14982 26466569

[165] Tutuka CSA Andrews MC Mariadason JM Ioannidis P Hudson C Cebon J. PLX8394, a new generation BRAF inhibitor, selectively inhibits BRAF in colonic adenocarcinoma cells and prevents paradoxical MAPK pathway activation. Mol Cancer. (2017) 16:112. doi: 10.1186/s12943-017-0684-x 28659148 PMC5490236

[166] Wichmann J Rynn C Friess T Petrig-Schaffland J Kornacker M Handl C. Preclinical characterization of a next-generation brain permeable, paradox breaker BRAF inhibitor. Clin Cancer Res. (2022) 28:770–80. doi: 10.1158/1078-0432.CCR-21-2761 34782366

[167] Piha-Paul SA Nagpal S Weise AM Braiteh FS Chen C Huang CQ. A phase 1, multicenter, open-label study of a new BRAF inhibitor ABM-1310 in adult patients (pts) with BRAFv600-mutated solid tumors. J Clin Oncol. (2023) 41:3098–8. doi: 10.1200/JCO.2023.41.16_suppl.3098

[168] Delphine L Luong H Maroua K Paul T Debra O Antonio DC. Meeting program and abstracts. Thyroid®. (2023) 33:P-1-A-124. doi: 10.1089/thy.2023.29161.lb.abstracts

[169] Montero-Conde C Ruiz-Llorente S Dominguez JM Knauf JA Viale A Sherman EJ. Relief of feedback inhibition of HER3 transcription by RAF and MEK inhibitors attenuates their antitumor effects in BRAF-mutant thyroid carcinomas. Cancer Discovery. (2013) 3:520–33. doi: 10.1158/2159-8290.CD-12-0531 PMC3651738 23365119

[170] Moretti S Menicali E Nucci N Guzzetti M Morelli S Puxeddu E. THERAPY OF ENDOCRINE DISEASE Immunotherapy of advanced thyroid cancer: from bench to bedside. Eur J Endocrinol. (2020) 183:R41–55. doi: 10.1530/EJE-20-0283 32449696

[171] French JD Kotnis GR Said S Raeburn CD McIntyre RC Jr. Klopper JP. Programmed death-1+ T cells and regulatory T cells are enriched in tumor-involved lymph nodes and associated with aggressive features in papillary thyroid cancer. J Clin Endocrinol Metab. (2012) 97:e934–943. doi: 10.1210/jc.2011-3428 PMC3387418 22466343

[172] Ding J Li D Liu X Hei H Sun B Zhou D. Chimeric antigen receptor T-cell therapy for relapsed and refractory thyroid cancer. Exp Hematol Oncol. (2022) 11:59. doi: 10.1186/s40164-022-00311-z 36138444 PMC9494903

[173] Edeline J Houot R Marabelle A Alcantara M. CAR-T cells and BiTEs in solid tumors: challenges and perspectives. J Hematol Oncol. (2021) 14:65. doi: 10.1186/s13045-021-01067-5 33874996 PMC8054411

[174] Li H Zhou X Wang G Hua D Li S Xu T. and potent preclinical activity against differentiated thyroid cancer. J Clin Endocrinol Metab. (2022) 107:1110–26. doi: 10.1210/clinem/dgab819 34751400

[175] Smallridge RC Copland JA. Anaplastic thyroid carcinoma: pathogenesis and emerging therapies. Clin Oncol (R Coll Radiol). (2010) 22:486–97. doi: 10.1016/j.clon.2010.03.013 PMC3905320 20418080

[176] Hamidi S Maniakas A. Recent advances in anaplastic thyroid cancer management. Curr Opin Endocrinol Diabetes Obes. (2023) 30(5):259–64. doi: 10.1097/MED.0000000000000823 37410453

[177] Chang CF Yang MH Lee JH Shih SR Lin CH Chen CP. The impact of BRAF targeting agents in advanced anaplastic thyroid cancer: a multi-institutional retrospective study in Taiwan. Am J Cancer Res. (2022) 12:5342–50. PMC9729886 36504909

[178] Lorimer C Cheng L Chandler R Garcez K Gill V Graham K. Dabrafenib and trametinib therapy for advanced anaplastic thyroid cancer - real-world outcomes from UK centres. Clin Oncol (R Coll Radiol). (2023) 35:e60–6. doi: 10.1016/j.clon.2022.10.017 36379836

[179] Zhao X Wang JR Dadu R Busaidy NL Xu L Learned KO. Surgery after BRAF-directed therapy is associated with improved survival in BRAF(V600E) mutant anaplastic thyroid cancer: A single-center retrospective cohort study. Thyroid. (2023) 33(4):484–91. doi: 10.1089/thy.2022.0504 PMC10122263 36762947

[180] Nazarian R Shi H Wang Q Kong X Koya RC Lee H. Melanomas acquire resistance to B-RAF(V600E) inhibition by RTK or N-RAS upregulation. Nature. (2010) 468:973–7. doi: 10.1038/nature09626 PMC3143360 21107323

[181] Bagheri-Yarmand R Busaidy NL McBeath E Danysh BP Evans KW Moss TJ. RAC1 alterations induce acquired dabrafenib resistance in association with anaplastic transformation in a papillary thyroid cancer patient. Cancers (Basel). (2021) 13:4950. doi: 10.3390/cancers13194950 34638434 PMC8507731

[182] Cabanillas ME Dadu R Iyer P Wanland KB Busaidy NL Ying A. Acquired secondary RAS mutation in BRAF(V600E)-mutated thyroid cancer patients treated with BRAF inhibitors. Thyroid. (2020) 30:1288–96. doi: 10.1089/thy.2019.0514 PMC7869871 32216548

[183] Danysh BP Rieger EY Sinha DK Evers CV Cote GJ Cabanillas ME. Long-term vemurafenib treatment drives inhibitor resistance through a spontaneous KRAS G12D mutation in a BRAF V600E papillary thyroid carcinoma model. Oncotarget. (2016) 7:30907–23. doi: 10.18632/oncotarget.v7i21 PMC5058727 27127178

[184] Duquette M Sadow PM Husain A Sims JN Antonello ZA Fischer AH. Metastasis-associated MCL1 and P16 copy number alterations dictate resistance to vemurafenib in a BRAFV600E patient-derived papillary thyroid carcinoma preclinical model. Oncotarget. (2015) 6:42445–67. doi: 10.18632/oncotarget.v6i40 PMC4767444 26636651

[185] Corcoran RB Ebi H Turke AB Coffee EM Nishino M Cogdill AP. EGFR-mediated re-activation of MAPK signaling contributes to insensitivity of BRAF mutant colorectal cancers to RAF inhibition with vemurafenib. Cancer Discovery. (2012) 2:227–35. doi: 10.1158/2159-8290.CD-11-0341 PMC3308191 22448344

[186] Brauner E Gunda V Vanden Borre P Zurakowski D Kim YS Dennett KV. Combining BRAF inhibitor and anti PD-L1 antibody dramatically improves tumor regression and anti tumor immunity in an immunocompetent murine model of anaplastic thyroid cancer. Oncotarget. (2016) 7:17194–211. doi: 10.18632/oncotarget.v7i13 PMC4941380 26943572

[187] Gunda V Gigliotti B Ashry T Ndishabandi D McCarthy M Zhou Z. Anti-PD-1/PD-L1 therapy augments lenvatinib's efficacy by favorably altering the immune microenvironment of murine anaplastic thyroid cancer. Int J Cancer. (2019) 144:2266–78. doi: 10.1002/ijc.32041 30515783

[188] Boudin L Morvan JB Thariat J Metivier D Marcy PY Delarbre D. Rationale efficacy and safety evidence of lenvatinib and pembrolizumab association in anaplastic thyroid carcinoma. Curr Oncol. (2022) 29:7718–31. doi: 10.3390/curroncol29100610 PMC9601195 36290887

[189] Hamidi S Iyer P Dadu R Gule-Monroe M Maniakas A Zafereo ME. Checkpoint inhibition in addition to dabrafenib/trametinib for BRAF(V600E) mutated anaplastic thyroid carcinoma. Thyroid. (2024) 34(3):336–46. doi: 10.1089/thy.2023.0573 38226606

[190] . National Comprehensive Cancer Network Guidelines - Thyroid Carcinoma. National Comprehensive Cancer Network. (2024) 1.2024.

[191] Lorch JH Barletta JA Nehs M Uppaluri R Alexander EK Haddad RI. A phase II study of nivolumab (N) plus ipilimumab (I) in radioidine refractory differentiated thyroid cancer (RAIR DTC) with exploratory cohorts in anaplastic (ATC) and medullary thyroid cancer (MTC). J Clin Oncol. (2020) 38:6513–3. doi: 10.1200/JCO.2020.38.15_suppl.6513

